# Track placement, school environments, and achievement gaps: identifying the drivers of the academic track advantage in Italy

**DOI:** 10.1186/s40536-026-00329-3

**Published:** 2026-10-30

**Authors:** Elisa Sbalchiero, Francesco Balzaretti, Emanuele Fedeli, Moris Triventi

**Affiliations:** 1 Graduate School in Social and Political Sciences, University of Milan “La Statale”, Milan, 20122 Italy; 2 Department of Social and Political Sciences, University of Milan “La Statale”, Milan, 20122 Italy

## Abstract

Educational tracking shapes students’ learning opportunities, yet isolating its effects from prior selection remains a key challenge in large-scale assessment research. This study examines both the size and the mechanisms of academic-track advantages by integrating longitudinal population-level administrative data from INVALSI with school- and student-level indicators from PISA 2018 in Italy. Focusing on lyceums versus technical schools, we exploit rich pre-tracking information on achievement, socio-demographic background, and early attitudes, and apply entropy balancing to adjust for selection into tracks. Results show that achievement gaps at Grade 10 are large across domains, but substantially reduced once differences in prior competencies and backgrounds are accounted for. Nonetheless, a meaningful academic-track advantage persists after balancing. Decomposition analyses indicate that this residual gap is largely explained by differences in school environments, particularly classroom academic and SES composition alongside school climate, while instructional practices and organizational policies or school resources contribute little on average. The findings support a dual interpretation of tracking effects, combining cumulative selection and exposure to differentiated learning contexts, and highlight the value of linking administrative panels with international assessment data to study both outcomes and mechanisms of educational stratification.

**Supplementary Information:**

The online version contains supplementary material available at https://doi.org/10.1186/s40536-026-00329-3.

## Introduction

Educational tracking, the systematic allocation of students into differentiated curricular pathways, remains one of the most consequential institutional features of secondary education worldwide (Van de Werfhorst & Mijs, 2010). By assigning students to academically or vocationally oriented routes, tracking structures the educational opportunities available to young people, with implications that extend far beyond the classroom. Research consistently shows that track placement shapes learning trajectories, influences educational transitions such as university enrolment, and affects long-term occupational outcomes (Brunello & Checchi, 2007; Borghans et al., 2019; Terrin & Triventi, 2023). Academic tracks typically offer broader and more demanding curricula, whereas vocational tracks emphasize applied training and early specialization. Consequently, the track students enter often shapes their subsequent life-course trajectories. Understanding how and why tracking matters is therefore crucial for debates on educational inequality, system design, and the extent to which schools can compensate for amplify pre-existing differences in skills and resources (Skopek et al., 2019). At the same time, estimating the impact of tracking on student learning is analytically challenging because track assignment is not random. Even when allocation is formally regulated through grades, teacher recommendations, or institutional rules, students entering different tracks may also differ in family resources, aspirations, information about educational options, and attitudes towards schooling. These additional social selection processes make it difficult to distinguish track effects from pre-existing differences between students (Blossfeld et al., 2016; Traini et al., 2021; Triventi et al., 2021).

These selection processes raise a first fundamental question that lies at the heart of international debates on tracking: Are the superior outcomes observed among academic-track students primarily the result of who these tracks attract, or do tracks themselves generate genuine learning advantages, further amplifying pre-existing achievement disparities?

This question underscores two interrelated research needs. First, we require credible causal estimates of track effects that convincingly separate genuine effects from pre-existing differences. Second, even when causal impacts are plausibly established, we must understand through which mechanisms these effects arise. Without identifying the channels that translate track placement into differential learning trajectories, it is difficult to judge which features of the academic tracks are potentially beneficial, which are harmful, and which could be reformed (Gamoran, 2009).

International scholarship identifies several channels through which tracking may affect achievement (Gamoran & Mare, 1989; Hallinan, 1994; Terrin & Triventi, 2023). Academic and vocational pathways differ in curriculum depth, subject focus, instructional demands, teacher allocation, peer environments, disciplinary climate, academic expectations, and expected post-secondary destinations (Hallinan, 1994; Maaz et al., 2008; Betts, 2011; Terrin & Triventi, 2023). These institutional and compositional differences shape both opportunities to learn and motivational cultures: higher-track students are often exposed to stronger peer performance, fewer behavioural disruptions, more cognitively demanding instruction, and a climate oriented towards academic progression, whereas lower-track environments may face resource constraints, weaker academic norms, and curricular limitations (Maaz et al., 2008; Traini et al., 2021). Yet evidence on the mechanisms underlying track effects remains incomplete, partly because much of the literature relies on cross-sectional data or controls only imperfectly for baseline differences (Skopek et al., 2019). As a result, estimated track effects often conflate treatment effects with selection bias, giving rise to a central methodological challenge that a substantial body of literature has sought to address (Triventi et al., 2021; Traini et al., 2021; Terrin & Triventi, 2023).

In parallel, a growing body of research, particularly in countries with early between-school tracking, documents persistent “scissor effects,” whereby achievement gaps between tracks widen over time, even among students entering secondary education with similar initial skills (Traini et al., 2021; Triventi et al., 2021). Yet the mechanisms driving this divergence remain contested, and relatively few studies have been able to empirically distinguish between competing explanations or assess their relative contributions.

Once genuine academic-track advantages are established, the subsequent question is which features of the school environment explain the resulting achievement gaps between tracks. Addressing this question requires datasets that combine (i) rich pre-tracking measures of students’ competencies, motivations, and socio-economic background; (ii) reliable post-tracking outcomes; and (iii) detailed indicators of school-level processes—including peer composition, disciplinary climate, teacher support, instructional practices, resource endowments, and student motivations. Such integrated data are rarely available in international research, where achievement measures (e.g., PISA) often lack pre-tracking baselines, while administrative datasets typically lack information on school environments.

The present study leverages a unique opportunity to advance this research agenda by linking PISA 2018 school-level indicators with rich longitudinal administrative data from INVALSI, covering Italian students from Grade 5 to Grade 10. Although Italy represents only one case within the broader set of early-tracking countries, it provides a theoretically important context for two reasons (Contini & Triventi, 2016). First, its clearly differentiated upper-secondary tracks make it an ideal setting for examining how qualitatively different school environments contribute to skill development. Second, the availability of population-level data on students’ prior competencies and backgrounds allows us to identify selection processes with far greater precision than is typically possible in international assessments.

By integrating these two data sources, we provide new evidence on both the magnitude of the academic-track advantage and the school-level mechanisms that contribute to it. We examine whether students enrolled in the academic track outperform their peers in other pathways even after accounting for extensive pre-tracking characteristics, and assess which dimensions of school environments explain any remaining differences. Specifically, we focus on four dimensions of the school learning environment: (1) classroom socioeconomic and academic composition; (2) school climate; (3) instructional practices and teacher support; and (4) school resources, policies, and activities.

Through this approach, the study contributes to longstanding debates on the consequences of tracking, the production of learning inequalities, and the extent to which schools compensate for or reinforce social stratification.

## Achievement differences by school tracks: previous studies

Tracking can take different institutional forms. In within-school tracking, students are allocated to ability groups or course levels while remaining in the same school; in between-school tracking, students enter institutionally distinct schools or programmes, often associated with different curricula, teacher profiles, peer environments, and post-secondary destinations (Betts, 2011; Blossfeld et al., 2016). The Italian case examined here corresponds to the latter form: after comprehensive lower-secondary education, students choose among distinct upper-secondary school types (Contini & Scagni, 2013). Accordingly, our review focuses primarily on between-school tracking, where track placement is likely to imply stronger differentiation in learning environments.

A large literature examines whether attending different secondary-school tracks shapes achievement. Descriptive studies, often based on international assessments, consistently show that students in academic tracks outperform those in technical or vocational tracks (Marks et al., 2006; Dronkers et al., 2011; OECD, 2016). However, because track placement is strongly patterned by prior achievement, social background, aspirations, and other pre-existing unobserved characteristics, such differences cannot be straightforwardly interpreted as track effects. Beyond descriptive comparisons, a closely related strand studies learning rates or “scissor effects”, asking whether achievement gaps between academic-track and non-academic-track students widen after track allocation. Evidence from Germany generally indicates stronger gains in cognitive skills or achievement among students in academic tracks than among comparable students in non-academic tracks (Becker et al., 2012; Guill et al., 2017; Pfost et al., 2010; Retelsdorf et al., 2012). Evidence of differential achievement trajectories has also been documented in other early or explicit tracking systems, including Hungary, Switzerland, and Flanders (Angelone, 2019; Bach et al., 2024; Dockx, de Fraine, and Vandecandelaere, 2019; Hermann, 2013; Horn, 2013).

Recent studies have made progress by combining longitudinal data with stronger adjustments for track selection. For Italy, Triventi et al. (2021) use population-level INVALSI data and a difference-in-differences design combined with marginal mean weighting and attrition correction. They show that selection explains a substantial part, but not all, of cross-track achievement gaps, with residual effects especially pronounced in mathematics and varying across curricula. This study is particularly relevant for our case because it examines the same national context and demonstrates that track differences cannot be reduced to pre-existing disparities alone. However, it does not directly assess which school-level features account for the residual advantage of academic tracks.

Traini et al. (2021) are especially close to our approach from a methodological and mechanism-oriented perspective. Using German NEPS data and entropy balancing, they compare students in upper and intermediate tracks and find higher learning progress in the upper track, particularly in reading. Moreover, their results suggest that average class performance—and, to a lesser extent, social background composition—accounts for part of the track-related divergence, whereas migration composition and student-reported instructional quality contribute little.

Taken together, these contributions conceptualize track differences as the joint product of pre-track sorting and post-track exposure to differentiated learning environments. Yet evidence on the mechanisms behind such exposure effects remains comparatively limited. Prior research points to curriculum intensity, peer and classroom composition, disciplinary climate, instructional quality, teacher allocation, and school resources or organizational practices as plausible channels (Hallinan, 1994; Maaz et al., 2008; Betts, 2011; Traini et al., 2021), but few studies quantify their relative contribution. Our study builds directly on this literature by integrating longitudinal population-level INVALSI data, which allow rich adjustment for pre-track selection, with PISA-based school-environment measures, which make it possible to examine which observed features of upper-secondary schools account for residual achievement gaps. In this respect, the paper extends Triventi et al. (2021) by opening the black box of school-level mechanisms in the Italian case, and complements Traini et al. (2021) by providing comparable evidence from a different between-school tracking system, a later stage of secondary education and considering a wider set of mediators.

## Theoretical framework

Building on the international evidence on track effects, a useful theoretical starting point is that the observed achievement advantage of the academic track reflects a combination of *selection and genuine track effects*. In most settings, academic-track students display higher test scores already *before* entering the track, and the gap often widens thereafter. This pattern is consistent with a process in which (i) students who are already advantaged are more likely to sort into the academic track, and (ii) the academic track subsequently provides learning environments that further promote achievement (Gamoran & Mare, 1989; Hanushek and Woessmann, 2006; Traini et al., 2021; Triventi et al., 2021).

### Selection into the academic track

Track placement is not random. Following Boudon’s (1974) distinction between primary and secondary effects of social origin, selection into academic tracks may reflect both earlier differences in achievement and socially stratified educational choices among students with similar prior performance. Primary effects operate through cumulative differences in competencies before track allocation, whereas secondary effects concern family resources, information, aspirations, risk perceptions, and expectations about the costs and returns of academic education. In addition, tertiary effects may arise from the institutional actors and organizational processes that translate students’ prior performance and family resources into educational placements, for instance through teacher recommendations, counselling practices, school-level expectations, or local opportunity structures (Esser, 2016). In formally open systems such as Italy, where teacher recommendations are non-binding, these channels are likely to operate jointly: prior achievement shapes academic eligibility and perceived suitability, families differ in their propensity to choose academically oriented pathways, and schools may contribute to the formation of expectations and recommendations that guide track choice (Contini & Triventi, 2016).

We now discuss each form of selection in turn, situating it within the broader literature and ongoing scholarly debate. *Social selection* reflects the stratification of educational choices by social origin. Students from upper-class and highly educated families are more likely to enter academic programs, even conditional on measured ability, because they possess greater informational resources, stronger educational norms, and a higher perceived payoff to academic credentials (Breen & Goldthorpe, 1997). These families also tend to be more effective in navigating institutional rules and informal gatekeeping, thereby translating resources into advantageous placements (Triventi et al., 2020).

*Academic selection* is typically grounded in prior competencies and performance signals. Earlier test scores, grades, and teacher evaluations shape track placement directly (through formal criteria) and indirectly (through parental and student expectations). Consequently, academic tracks concentrate students with higher baseline achievement, which mechanically inflates cross-track achievement differences if pre-track performance is not fully accounted for (Blossfeld et al., 2016).

*Socio-emotional* *selection* concerns motivational and attitudinal factors that are unequally distributed across social groups and strongly predictive of academic-track enrolment. Aspirations, intrinsic motivation, self-concept, and positive attitudes toward school increase both the likelihood of choosing (or persisting in) academic tracks and the propensity to invest in learning. These factors are partly shaped by family background and prior schooling experiences, and they may remain imperfectly observed in many datasets, leading to residual selection even in rich empirical models (Traini et al., 2021; Triventi et al., 2021).

*School-related selection* further amplifies stratification. Prior academic experiences can shape entry into upper-secondary tracks through peer- and resource-related mechanisms that operate before tracking. Students educated in lower-secondary schools with stronger instructional resources and higher-achieving, academically oriented peer milieus are more likely to internalize pro-school norms, receive better information about educational pathways, and develop higher aspirations and academic self-concepts—factors that increase the likelihood of choosing the academic track (Jackson, 2013; Blossfeld et al., 2016)

### Tracking, opportunities to learn, and school-level mechanisms

Once students enter distinct tracks, achievement differences may widen because tracks provide different opportunities to learn. From this perspective, track effects operate through differences in the amount, quality, and organisation of learning opportunities available to students (Carroll, 1963; Hallinan, 1994; Rowan & Correnti, 2009). We distinguish four theoretically motivated domains: (1) classroom socioeconomic and academic composition captures the peer context in which students learn; (2) school climate, that captures the behavioural and normative conditions of learning; (3) instructional quality, which concerns the organisation and delivery of teaching; (4) school policies, resources, and activities that capture organisational features that structure learning opportunities. *Classroom academic and socioeconomic composition.* The first domain concerns classroom composition. In between-school tracking systems, selection into academic and technical pathways does not only allocate individual students to different curricula; it also produces classrooms that differ systematically in average prior achievement and socioeconomic background. Academic tracks tend to concentrate students with stronger prior competencies and more advantaged family resources, thereby creating environments characterized by higher average academic preparation, stronger school-oriented norms, and greater familiarity with institutional expectations.

Classroom academic composition may affect achievement by facilitating knowledge diffusion, raising the pace and level of instruction, providing academic role models, and reinforcing norms of effort and achievement. These mechanisms are consistent with social learning perspectives, which emphasize that students learn not only from teachers but also by observing and interacting with more competent peers (Bandura, 1977, 1986). At the same time, high-achieving peer contexts may have ambivalent effects if upward comparisons reduce academic self-concept, as suggested by social comparison theory and the big-fish-little-pond literature (Festinger, 1954; Davis, 1966; Marsh, 1987; Dicke et al., 2018). We therefore treat classroom average prior achievement not as evidence of a single peer mechanism, but as a broad indicator of the academic composition of the learning environment, capturing the level of preparation, instructional pace, and peer norms to which students are exposed.

Classroom socioeconomic composition captures a related but distinct dimension of the peer environment. Students from more advantaged families often bring stronger academic orientations, greater familiarity with school expectations, and more information about educational progression. Socioeconomic composition may therefore shape achievement through peer norms, teachers’ expectations, classroom behaviour, instructional pace, and the availability of academically relevant information and support among classmates (Willms, 2010; Chzhen & Leesch, 2023). Prior research on tracking has emphasized that peer achievement and social-background composition are central to the production of differential learning environments across tracks (Maaz et al., 2008; Hattie, 2002; Traini et al., 2021). We therefore expect classroom academic and socioeconomic composition to account for a substantial part of the residual academic-track advantage, while interpreting their contribution as compositional rather than purely institutional.

*School climate.* A second domain concerns school climate, understood here as the behavioural and normative conditions that structure everyday learning in schools and classrooms. This includes the extent to which lessons take place in an orderly environment, behavioural disruptions are limited, and academic engagement is socially supported. Tracking may affect school climate by sorting students into more homogeneous instructional settings, thereby shaping discipline, classroom order, and the extent to which effort and compliance are socially rewarded. A more stable and orderly climate can increase effective instructional time and support higher learning rates (Maaz et al., 2008; Kutsyuruba et al., 2015). In more academically oriented settings, stronger norms of effort and compliance may also foster diligence, persistence, and sustained investment in school tasks. We therefore expect academic tracks to exhibit more favourable disciplinary and behavioural climates, which may contribute to higher achievement.

*Instructional quality and teaching practices.* School tracks may also differ in curriculum focus, working conditions, and pedagogical traditions (Schiepe-Tiska, 2019). These differences can shape instructional quality, including teacher support, classroom management, adaptive teaching, teacher–student relations, and teacher enthusiasm (OECD, 2018). High-quality instruction is widely recognized as a central determinant of student achievement (Weinert et al., 1989), and teachers’ pedagogical content knowledge, professional beliefs, and motivation may influence learning through cognitive activation and individualized support (Baumert et al., 2010; Kunter et al., 2013). In Italy, differences in instructional quality may also reflect teacher allocation across tracks: teachers in academic tracks tend to be older and more often permanently employed, while younger and non-permanent teachers are more frequently found in other school types (Bryson et al., 2022). This pattern may arise because senior teachers with permanent contracts have greater priority in school choice and may self-select into schools perceived as offering better working conditions (Abbiati et al., 2017).

*Material resources and organizational policies.* Finally, material resources and organizational policies may contribute to track-specific learning environments. Where academic and non-academic pathways are housed in distinct institutions, track placement may expose students to different levels of material resources, staffing, instructional organisation, and opportunities for cognitively demanding learning (Maaz et al., 2008; Betts, 2011; Traini et al., 2021). If such differences exist, they may structure unequal learning opportunities and contribute to achievement differences, although whether they play an independent role remains an empirical question (Brunello & Checchi, 2007). Relevant dimensions include class size (Wößmann, 2005), quality assurance practices (Wößmann, 2007), cultural and extracurricular activities (Karakus et al., 2023), ability grouping within schools (Tan & Dimmock, 2022), and additional reading instruction outside regular school hours (OECD, 2018).

Based on the Opportunity to Learn framework and the institutional logic of tracking, we expect academic-track schools to provide more favourable learning environments than non-academic schools. Specifically, we expect them to differ in classroom academic and socioeconomic composition, school climate, instructional quality, and organizational resources and policies. To the extent that these dimensions differ across tracks and are associated with achievement, they should account for part of the adjusted academic-track advantage.

It is important to note that these dimensions are partly institutional and partly compositional. Institutional channels derive from the formal organisation of tracks, including curricula, staffing, resources, and school policies. Compositional channels arise from differences in the characteristics of the student body across schools offering different tracks. Some dimensions, especially school climate, likely reflect both processes: they may be shaped by institutional arrangements, but also by the characteristics, norms, and interactions between students and teachers. Although analytically distinct, these domains are therefore expected to interact and jointly shape the learning environments experienced by students across educational tracks.

## Key features of the Italian tracking system

Italy is characterized by late but consequential tracking. Students attend a fully comprehensive system through lower secondary education (Grade 8, typically age 14) and then choose among three main upper-secondary branches: lyceums (licei), technical schools (istituti tecnici), and vocational schools (istituti professionali) (Contini & Scagni, 2013; Contini & Triventi, 2016). Formal ability-based admission is not allowed and teacher recommendations are non-binding. The choice is therefore formally open, yet strongly socially and academically structured: students from more advantaged families, with higher prior achievement, and with stronger motivation, diligence, and attachment to school are disproportionately channelled into lyceums (Contini & Scagni, 2013; Panichella & Triventi, 2014; Contini & Triventi, 2016; Triventi et al., 2021).

From a substantive perspective, the most relevant contrast for this study is between lyceums and technical schools. These two branches enrol large shares of students and both preserve meaningful access to university, while differing clearly in curricular orientation and expected trajectories (Contini & Scagni, 2013; Contini & Triventi, 2016). Technical schools represent an intermediate option: they combine occupationally oriented curricula with a relatively stronger emphasis on general competencies and show substantially higher rates of university entry and completion than vocational schools. Vocational schools, by contrast, display more marked negative selection and weaker post-compulsory trajectories (Contini & Scagni, 2013; Checchi & Flabbi, 2013; Contini & Triventi, 2016).

A further reason for sharpening the treatment definition concerns heterogeneity within the academic branch. Alongside the historically most prestigious and demanding curricula—classical and scientific lyceums—Italy hosts other lyceums, such as language, arts, and human-sciences programmes, which are institutionally academic but less clearly aligned with the traditional university-preparatory model. Because curricular emphases, especially in mathematics, differ substantially across these options, pooling all lyceums would blur the meaning of the academic-track treatment. We therefore focus on classical and scientific lyceums versus technical schools, a comparison that captures a substantively important “at-the-margin” choice while maintaining a clearer treatment definition.

## Analytical strategy

### Data sources

To study the achievement consequences of upper-secondary tracking and the role of school environments, we combine two complementary data sources: (i) population-level administrative and assessment data from INVALSI-SNV, and (ii) the Italian national sample of OECD–PISA 2018, which provides rich information on school climate and organizational features. The integration is designed to leverage the respective strengths of each source.

The first source is represented by data collected by INVALSI (*Istituto Nazionale per la Valutazione del Sistema Educativo di Istruzione e di Formazione*, National Institute for the Evaluation of the Education and Training System), which administers standardized assessments to the full population of students enrolled in selected grades in each school year (*Sistema Nazionale di Valutazione SNV*, Italian National Evaluation System). INVALSI-SNV data are particularly well suited to our research aims for various reasons. First, they provide high-quality measures of student achievement in language (Italian) and mathematics based on standardized tests administered under common procedures across schools. These tests are designed to capture competencies related to curriculum-specific knowledge and that extend beyond it, being relevant for adult life and labor-market participation. Second, INVALSI combines test scores with rich background information derived from school registers and from an ad hoc student questionnaire administered in selected grades (notably Grade 5 and Grade 10). Moreover, they provide anonymized classroom and school identifiers (IDs). This enables us to measure, prior to track entry, a rich set of predictors of educational choice and achievement. Third, because the data cover the entire population, estimates are not affected by sampling variability and can be obtained with high precision, which is particularly valuable for documenting selection patterns and estimating residual gaps after extensive balancing.

The second dataset is derived from the Programme for International Student Assessment (PISA), a cross-national survey coordinated by the OECD that assesses the competencies of 15-year-old students every three years (OECD, 2019). PISA uses a two-stage stratified sample design: schools are first sampled with probability proportional to size, and then students are randomly sampled within participating schools. The survey combines (i) standardized competency tests and (ii) extensive background questionnaires administered to students, together with a school principal questionnaire collecting information on institutional characteristics and organizational practices. PISA 2018 offers two main advantages for our purposes. First, it provides rich, internationally standardized measures of school climate and organizational practices, constructed using common instruments and coding procedures. Second, the combination of student and principal questionnaires yields school-level information on instructional processes, policies, and resources that is largely unavailable in administrative archives, thereby enabling a more direct empirical assessment of potential mechanisms behind track-related achievement gaps. At the same time, PISA has important limitations when used as a stand-alone source. As a sample survey, it covers a smaller number of students than population-based data such as INVALSI. Moreover, it is cross-sectional at age 15 (approximately Grade 10), which constrains the possibility of addressing selection into tracks using detailed pre-track students’ histories.

### Data integration

**Fig. 1:**
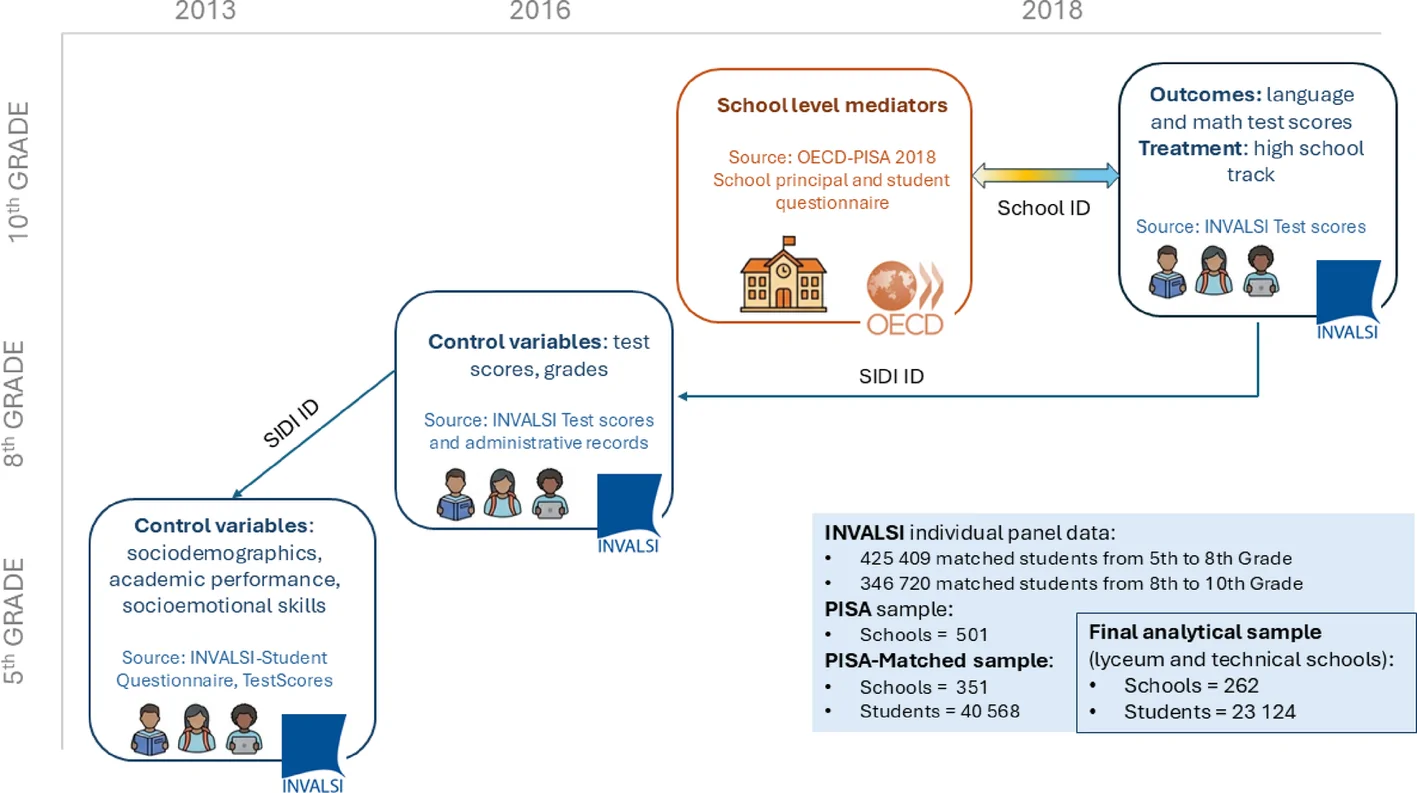
Illustration depicting the main data sources and the construction of the analytical sample

We constructed the analytical dataset in two steps. First, we built an INVALSI longitudinal panel by exploiting the unique student identifier available in INVALSI archives, which allows individuals to be followed across testing waves. Specifically, we link students assessed in Grade 5 (2012/13) to the same individuals observed three years later in Grade 8 (2015/16) and five years later in Grade 10 (2017/18). This procedure yields a panel of 425,409 students matched from Grade 5 to Grade 8 and 346,720 students matched from Grade 8 to Grade 10 (Fig. 1). The resulting panel provides pre-track covariates and prior achievement (Grades 5 and 8), together with outcomes measured after entry into upper secondary education (Grade 10).

Second, we integrated PISA 2018 information by matching at the school level. We link PISA school identifiers to the corresponding INVALSI school code (SIDI ID) and then assign PISA-based school measures—capturing classroom composition, school climate, instructional processes, and school policies/resources—to INVALSI Grade-10 student records through the matched school. In other words, PISA contributes school-level mediators measured in upper secondary education, while INVALSI contributes to population-level student outcomes and detailed pre-track students’ histories.

This matching procedure produces a multi-stage analytical sample. The Italian PISA 2018 dataset includes 501 schools. After merging PISA and INVALSI via school identifiers, the integrated dataset contains 351 matched schools and 40,568 Grade-10 students with INVALSI outcomes and linked PISA school information. Finally, to focus on the substantively central comparison in the Italian context and to maximize comparability of curricula and destinations, we restrict the analysis to students enrolled in lyceums and technical schools, resulting in a final sample of 262 schools and 23,124 students. Additional matching diagnostics indicate that the linked analytical sample remains highly comparable to the broader INVALSI population on key student-level characteristics, including prior achievement, socioeconomic background, gender, and birth cohort. At the school level, matched and unmatched schools are also similar in average achievement and student composition, although larger schools are, as expected, overrepresented because of PISA’s school-sampling design. See Online Appendix (Tables A3 and A4) for more detailed information.

A key advantage of our integration strategy is that the linkage is performed at the school level, which allows us to attach PISA-derived school measures to a comparatively large number of students observed in INVALSI. This preserves the main strengths of INVALSI—large sample size and longitudinal pre-track histories—while incorporating the otherwise unavailable information on school climate and practices needed to study potential mechanisms behind the lyceum advantage.

### Variables

*Outcomes.* The outcome variables are students’ reading and mathematics achievement in Grade 10, measured using INVALSI standardized tests administered to upper secondary school students. INVALSI applies a Rasch item response theory (IRT) model and derives achievement scores using Weighted Likelihood Estimation (WLE) (Desimoni, 2018). The reading test assesses grammatical knowledge and text comprehension, while the mathematics test measures reasoning and computational skills. Test scores were standardized to have a mean of 0 and a standard deviation of 1.

The *treatment variable* captures upper-secondary track placement. We distinguish students enrolled in the classical and scientific lyceum from those enrolled in technical schools, and we restrict the analytical sample to these two tracks. We restrict the academic option to the classical and scientific lyceum because “lyceums” are internally heterogeneous (e.g., arts, languages, human sciences) in curricular intensity and post-secondary prospects; pooling them would blur the treatment definition, especially for mathematics. Students in other lyceums and vocational streams are therefore excluded (coded as missing) in the main analysis.

*Control variables.* The student-level covariates considered, spanning Grades 5 to 8, include prior academic achievement in Grades 5 and 8, measured using standardized Italian language and mathematics test scores, as well as a set of socio-demographic characteristics. These include student ESCS (Grade 5),^1^ migration background (Grade 5), sex (based on information from Grades 5 and 8), geographical area of residence (using measures from both Grades 5 and 8), and number of siblings (Grade 5). In addition, several indices and variables measuring students’ attitudes are drawn from Grade 5, including reading and mathematics enjoyment, academic diligence, extrinsic motivation, and test anxiety. Finally, an indicator of students’ educational status is taken from Grade 8. These covariates are used to render the treatment and control groups comparable by reweighting units through the entropy balancing procedure described below. Together, these covariates capture the main pre-treatment dimensions expected to influence both track choice and later achievement, thereby reducing observable selection bias in the lyceum–technical comparison.

Table 1 provides a description of the outcome variables, the treatment variable, and the covariates drawn from INVALSI data used in the analysis, including the school-level covariates aggregated from the Italian administrative data (average classroom ESCS and classroom average prior achievement in language and mathematics). The next section describes the school-level covariates derived from the PISA 2018 School and Student Questionnaires.

**Table 1:** Description of the individual-level outcomes and treatment variable, the school-level variables, and the control variables used for entropy balancing, based on INVALSI data

Variable	Description	Source
Outcomes		
Reading achievement in Grade 10	Standardized test scores in the INVALSI Italian language assessment	INVALSI Student Questionnaire (Grade 10) 2017/2018
Math achievement in Grade 10	Standardized test scores in the INVALSI mathematics assessment	INVALSI Student Questionnaire (Grade 10) 2017/2018
Treatment		
Educational track placement	Dichotomous variable distinguishing among lyceum and technical tracks	INVALSI Student Questionnaire (Grade 10) 2017/2018
Classroom level mediators		
Average classroom ESCS	Classroom average ESCS score, measuring the average socioeconomic background of students in the classroom through an index combining parental education, parental occupational status, and educational and cultural resources available at home.	INVALSI Student Questionnaire (Grade 10) 2017/2018
Average classroom prior language achievement (Grade 8)	Classroom average prior language achievement, measured as the mean Grade 8 INVALSI language achievement score of students in the classroom. The variable captures the average prior achievement of classmates before entry into upper secondary education.	INVALSI Student Questionnaire (Grade 8) 2015/2016
Average classroom prior mathematics achievement (Grade 8)	Classroom average prior mathematics achievement, measured as the mean Grade 8 INVALSI mathematics achievement score of students in the classroom. The variable captures the average prior achievement of classmates before entry into upper secondary education.	INVALSI Student Questionnaire (Grade 8) 2015/2016
Student level covariates		
Reading achievement in Grade 8	Standardized test scores in the INVALSI Italian language assessment	INVALSI Student Questionnaire (Grade 8) 2015/2016
Math achievement in Grade 8	Standardized test scores in the INVALSI mathematics assessment	INVALSI Student Questionnaire (Grade 8) 2015/2016
Reading achievement in Grade 5	Standardized test scores in the INVALSI Italian language assessment	INVALSI Student Questionnaire (Grade 5) 2012/2013
Math achievement in Grade 5	Standardized test scores in the INVALSI mathematics assessment	INVALSI Student Questionnaire (Grade 5) 2012/2013
Student ESCS	Continuous index constructed by combining information on parental education, parental occupational status, and the educational and cultural resources available at home.	INVALSI Student Questionnaire (Grade 5) 2012/2013
Migration background	Derived indicator that combines information on the student’s and parents’ countries of birth. Students are classified as native, second-generation immigrant, or first-generation immigrant.	INVALSI Student Questionnaire (Grade 5) 2012/2013
Student sex	Dichotomous variable distinguishing between male and female students.	INVALSI Student Questionnaire Derived from Grade 5 and Grade 8 information (2012/2013–2015/2016)
Geographical area of residence in Grade 8	Defined using the standard Italian macro-regional classification, distinguishing between North-West, North-East, Centre, South, and Islands.	INVALSI Student Questionnaire (Grade 8) 2015/2016
Geographical area of residence in Grade 5	Defined using the standard Italian macro-regional classification, distinguishing between North-West, North-East, Centre, South, and Islands.	INVALSI Student Questionnaire (Grade 5) 2012/2013
Reading appreciation in Grade 5^1^	Standardized index measuring students’ enjoyment of reading activities.	INVALSI Student Questionnaire (Grade 5) 2012/2013
Mathematics appreciation in Grade 5	Standardized index measuring interest and positive attitudes toward mathematics.	INVALSI Student Questionnaire (Grade 5) 2012/2013
Academic diligence in Grade 5	Standardized indicator measuring students’ effort, persistence, and engagement in school tasks.	INVALSI Student Questionnaire (Grade 5) 2012/2013
External motivation in Grade 5	Standardized index capturing the extent to which students are motivated by external rewards or recognition.	INVALSI Student Questionnaire (Grade 5) 2012/2013
Test anxiety in Grade 5	Standardized index measuring stress and worry related to assessment situations.	INVALSI Student Questionnaire (Grade 5) 2012/2013
Student status in Grade 8	Categorical variable measuring students’ progression through the school system, distinguishing between students who are on time for their grade and those who are delayed or have experienced grade repetition.	INVALSI Student Questionnaire (Grade 8) 2015/2016

*School-level mediators.* Several batteries of items from the PISA School Questionnaire, as well as student-level information aggregated to the school level, are used to construct school indices capturing characteristics of the school environment and the instructional context. The school-level variables capture school climate, instructional quality, and organizational policies. In addition to the PISA-derived school-level measures, the INVALSI data provide further variables capturing classroom composition. Specifically, we include average classroom ESCS, which captures the socioeconomic composition of the classroom, and average classroom prior achievement in language and mathematics, both constructed from INVALSI data and therefore presented in Table 1. Table 2 provides a description of the constructed variables and indices, along with their specific data source and Cronbach’s alpha coefficients.

**Table 2:**
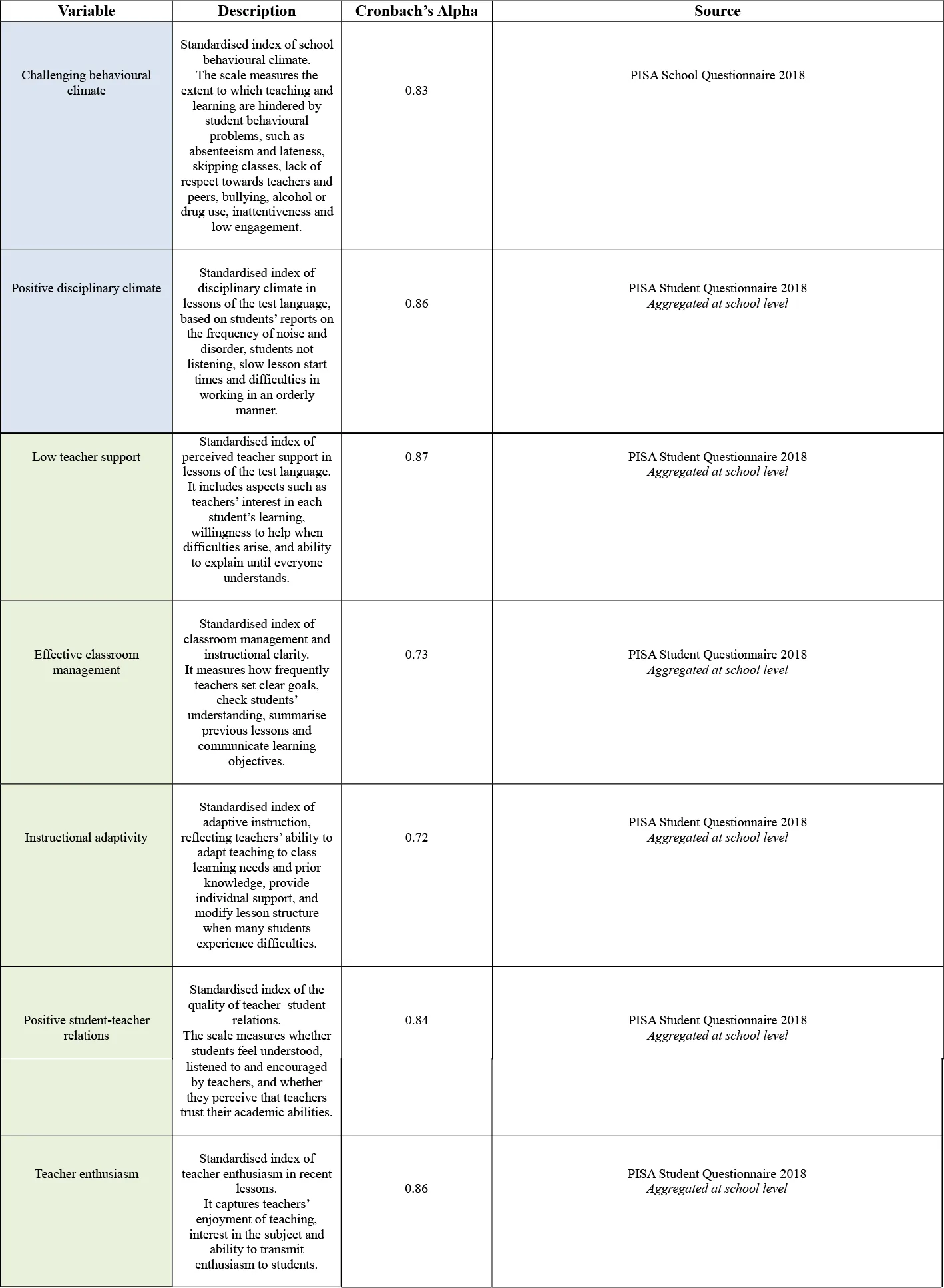

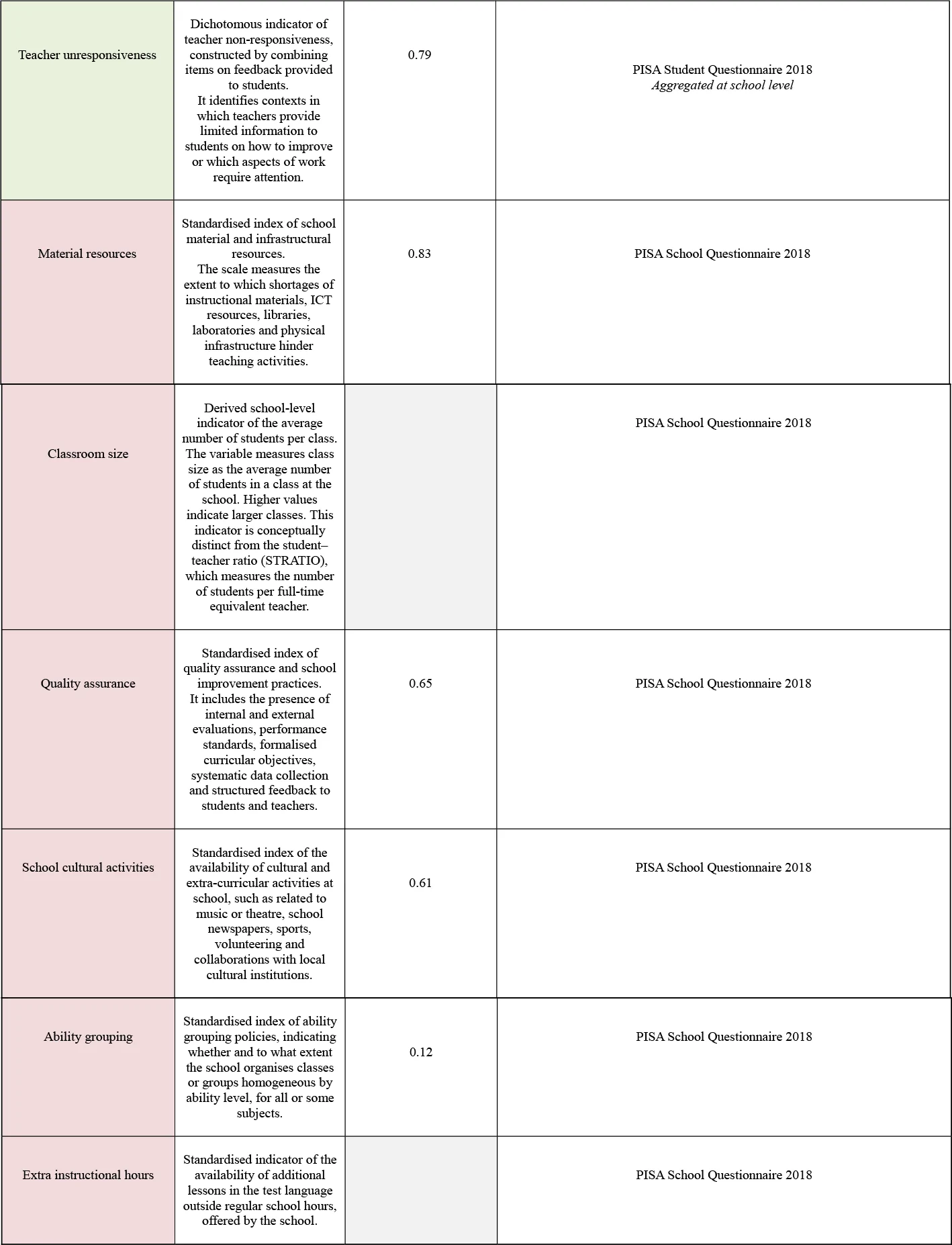
Description of school-level mediator variables and constructed indices derived from the PISA 2018 School and Student Questionnaires (Note: Blue squares indicate constructs belonging to the school climate-related block; green squares indicate those belonging to the instructional quality-related block; red squares indicate those belonging to the school resources and organizational policies-related block)

### Estimands, identification, and estimation methods

Our first theoretical estimand (Lundberg et al., 2021) concerns the average total effect of attending a lyceum (versus a technical school) on language and mathematics achievement on Grade 10 achievement, which takes the following form:

$$\tau ^{s} = {\mathbb{E}}\left[ {Y^{s} \left( 1 \right) - Y^{s} \left( 0 \right)} \right],s \in \left\{ {{\mathrm{lang}},{\mathrm{math}}} \right\}$$

Where s ∈ {lang, math} indexes the subject assessed (language or mathematics), and Y_s_(1) and Y_s_(0) denote a student’s potential grade-10 achievement in subject s under lyceum and technical-school attendance, respectively.

To approximate the corresponding empirical estimand, we use entropy balancing to construct weights that reweight one track group to match the other on the first three moments (mean, variance, skewness) of a rich set of pre-treatment covariates* X* (Hainmueller & Xu, 2013). In the resulting weighted pseudo-population, the empirical estimand is the difference in weighted mean outcomes:

$$\theta ^{s} = {\mathbb{E}}_{w} [Y^{s} {\mid }D = 1] - {\mathbb{E}}_{w} [Y^{s} {\mid }D = 0],$$

Where D indicates track attendance (D = 1 for lyceum and D = 0 for technical school) and E*w* denotes the expected estimate under the entropy-balancing weights.

The weighted achievement difference between lyceum and technical-track students is estimated using a weighted regression of academic achievement (${Y}^{s}$) on educational track placement (*D*):

$$Y_{i}^{s} = \alpha ^{s} + \tau ^{s} D_{i} + \varepsilon _{i}^{s} ,$$

The coefficient $\hat{\tau }^{s}$ (or the equivalent weighted mean difference) is the estimate of $\:{\theta\:}^{s}$, interpretable as the average treatment effect on the treated (ATT).

Entropy balancing is used to adjust for observable selection into tracks based on a rich set of pre-treatment individual characteristics. In the weighted regression, we additionally condition on a parsimonious set of key individual covariates—students’ ESCS, migration background, gender, and Grade-8 achievement in language and mathematics. Including these covariates in the outcome models accounts for any remaining residual differences in core pre-treatment characteristics after weighting, can improve precision and reduce sensitivity to minor imbalance or functional-form misspecification in either step. We do not balance on characteristics of receiving upper-secondary schools, since these are part of the differentiated learning environments whose contribution we aim to assess. Instead, we balance on rich individual pre-track characteristics and then examine how school-level mediators account for the remaining achievement gap.

Compared to traditional approaches such as regression adjustment or propensity score analysis, the use of the entropy balancing approach guarantees (a) covariate balance by construction, reducing errors in specification searching and making the adjustment objective transparent; (b) less model dependence and stronger finite-sample performance; (c) efficient use of data, avoiding ad hoc pruning while providing clear diagnostics via the weight distribution (Hainmueller, 2012). Interpreting $\:{\theta\:}^{s}\:$as informative about $\:{\tau\:}^{s}$requires a number of identification assumptions, which are typical of identification of causal effects with observational data: (i) consistency/SUTVA (each student’s observed outcome equals the potential outcome under their realized track, and no interference across units), (ii) conditional exchangeability given pre-treatment $\:X\:$(no unmeasured confounding of track choice and outcomes after conditioning on * X*), and (iii) positivity/overlap (each covariate profile in the target population has a non-zero probability of attending either track). Because our treatment is defined as exposure to a track-specific school environment, peer composition and school climate are treated as part of the exposure rather than as violations of the estimand. The no-interference assumption is therefore understood more narrowly as excluding spillovers across students assigned to different school environments, not peer processes within those environments.

In a second step, we seek to identify the plausible mechanisms underlying the average total effect of attending a lyceum versus a technical school. We do so by assessing the extent to which the remaining (post-balancing) lyceum–technical achievement gap can be accounted for by various school characteristics, grouped into four broad categories: (a) school composition and peers, (b) school climate, (c) pedagogical approaches and teaching quality, and (d) human and material resources.

To this aim, we implement a two-fold Kitagawa–Oaxaca–Blinder (KOB) decomposition of the weighted outcome differential using school-level characteristics as mediators. Let $\:G\in\:\left\{A,B\right\}\:$ index groups (e.g., * A=* lyceum, * B=* technical), $\bar{Z}_{A} ,\bar{Z}_{B}$ be group means of mediators and $\:{\beta\:}^{*}$ be a reference coefficient vector (commonly from a pooled model). The two-fold decomposition is:

$$R^{s} \equiv \bar{Y}_{A}^{s} - \bar{Y}_{B}^{s} = \underbrace {{(\bar{Z}_{A} - \bar{Z}_{B} )\beta ^{*} }}_{{{\text{Explained (quantity/endowments)}}}} + \underbrace {{[\bar{Z}_{A} (\beta _{A} - \beta ^{*} ) + \bar{Z}_{B} (\beta ^{*} - \beta _{B} )]}}_{{{\text{Unexplained (coefficients/returns + residual)}}}}\;.$$

The decomposition partitions the weighted gap into: (*i*) an explained component attributable to between-track differences in observed mediator levels (endowments), and (*ii*) an unexplained component attributable to differences in coefficients (returns) and residual factors. We use the pooled option, which sets the reference coefficients to those from a pooled model including a group indicator and is recommended for two-fold decompositions (Jann, 2018). Given the large number of mediators and the potential endogeneity among several of them (e.g., school climate and school sociodemographic composition), we estimated the models either by including all mediators simultaneously or by grouping them into theoretically defined blocks for the decomposition analysis. From these models, we extract two quantities of interest. First, we estimate the share of the explained component attributable to each of the four theoretically defined mediator blocks. Second, we estimate the contribution of each specific school-level mediator to the explained component of the lyceum–technical achievement gap. The school-level characteristics considered here should be interpreted as properties of the learning environments experienced by students in different tracks. Although the adjusted achievement gap is estimated after balancing students on rich pre-tracking characteristics, the subsequent decomposition is descriptive rather than causal. Accordingly, our aim is not to isolate the causal effect of individual mediators, but to identify which dimensions of the school environment are most strongly associated with the adjusted lyceum–technical achievement gap.

## Empirical findings

### Selection into Lyceum vs. Technical tracks

**Fig. 2:**
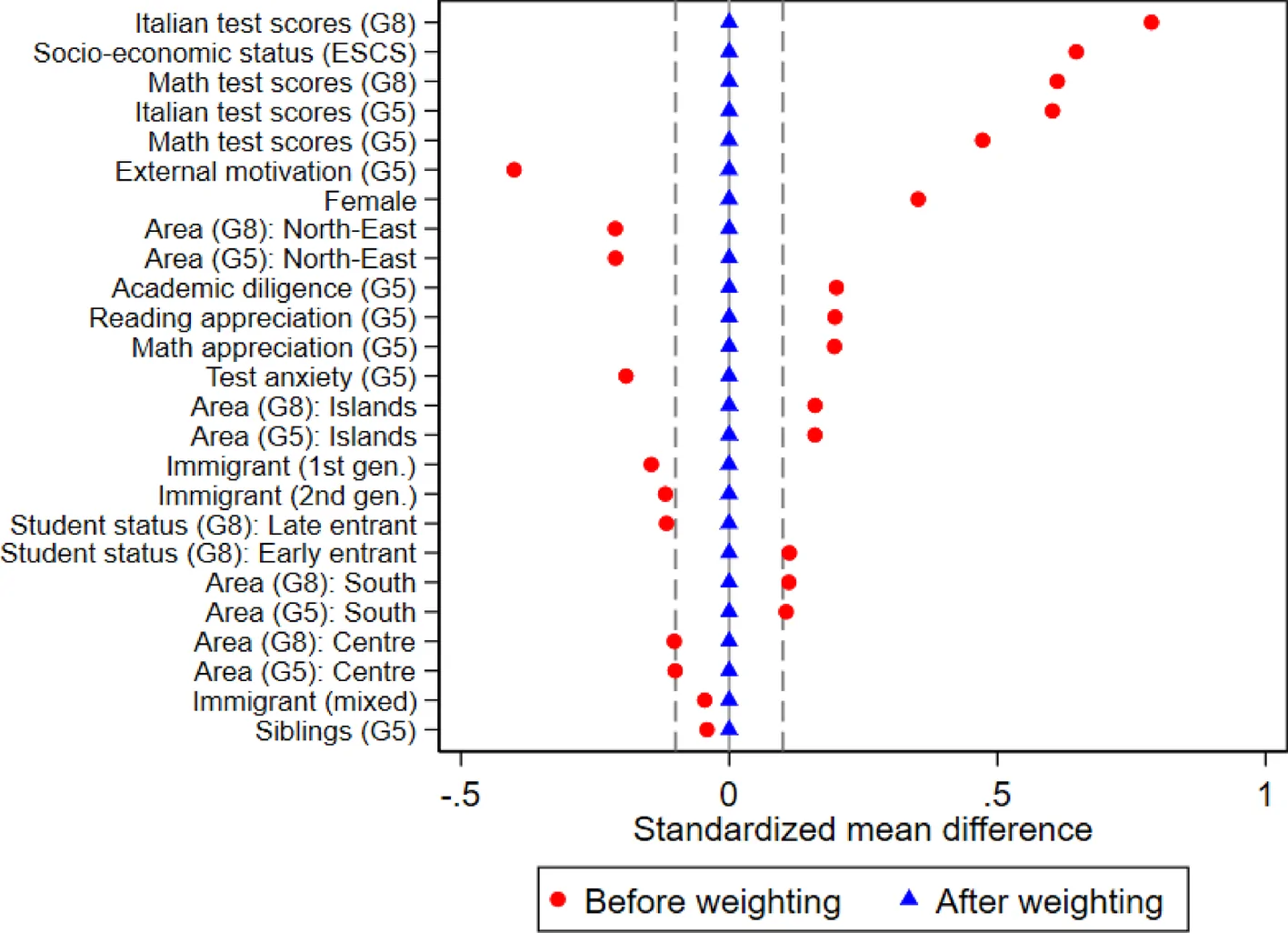
Balancing plot of pre-tracking individual-level characteristics across lyceums versus technical schools: before and after entropy-balancing weights

To assess the extent of selection into upper-secondary tracks, we examine standardized mean differences (SMDs) in pre-treatment characteristics between lyceum and technical students. The SMD for a covariate *X* is defined as the difference in group means divided by a pooled standard deviation, ${\mathrm{SMD}} = \left( {\bar{X}_{{{\mathrm{lyc}}}} - \bar{X}_{{{\mathrm{tech}}}} } \right)/s_{p}$. This metric is scale-free and directly interpretable in standard deviation units: values close to zero indicate good balance, while larger absolute values indicate stronger imbalance. In applied work, absolute SMDs below about 0.10 are often taken as a rule-of-thumb for negligible imbalance, whereas values above 0.25–0.50 suggest substantial selection. In Fig. 2 we compare the SMDs in the unweighted sample and in the weighted sample relying on the weights provided by entropy balance, to assess the degree of success in our strategy of making lyceums and technical schools similar on observed pre-tracking students’ characteristics.

The pre-weighting SMDs indicate pronounced selection into lyceums along both prior achievement and socioeconomic background dimensions. In particular, lyceum students score markedly higher already in earlier grades: the raw differences are large for grade-5 test scores (Italian: 0.60 SD; math: 0.47 SD) and even larger by grade 8 (Italian: 0.79 SD; math: 0.61 SD). Likewise, the socioeconomic index (ESCS) is substantially higher among lyceum students (0.65 SD), confirming strong social stratification in track entry.

Selection is also visible for other pre-treatment traits. Lyceums enroll more girls (SMD 0.35), and they are characterized by more favorable attitudinal profiles: higher reading appreciation (0.20), mathematics appreciation (0.20), and academic diligence (0.20). Conversely, students in technical schools appear more extrinsically motivated (SMD − 0.40) and exhibit higher test anxiety in early school stages (SMD − 0.19), suggesting that the two tracks attract students with systematically different orientations toward school and evaluation.

Geographic imbalances are non-trivial as well, with several area-of-residence indicators showing SMDs around 0.10–0.21 in absolute value, implying that track allocation also varies by place of residence and local opportunity structures. By contrast, migration background indicators show comparatively smaller differences (approximately − 0.05 to − 0.15) and the number of siblings is near-balanced (SMD − 0.04). Finally, educational pathway/status indicators also differ modestly in both directions (around ± 0.11), consistent with early differentiation in trajectories prior to the upper-secondary transition.

Overall, these patterns suggest that the raw lyceum–technical achievement gap might conflate track exposure with substantial pre-existing differences in academic performance, social origin, and motivational profiles—precisely the kinds of selection mechanisms emphasized by the literature.

After applying entropy-balancing weights (matching the first three moments of the covariate distributions), all SMDs shrink to values that are virtually zero (on the order of $\:{10}^{-4}$ or smaller). This indicates that the weighted comparison achieves an extremely high degree of covariate balance across the observed pre-treatment characteristics, thereby reducing model dependence and improving the plausibility of the subsequent adjusted comparisons of outcomes.

### Similarities and differences in the learning environments between lyceums and technical schools

Before turning to the decomposition analysis, we first examine whether the school-environment characteristics considered as potential mediators are unequally distributed across tracks. This step is important because a school characteristic can contribute to explaining the lyceum–technical achievement gap only if students in the two tracks are exposed to different levels of that characteristic and if the characteristic is associated with achievement.

Table 3 reports descriptive statistics and standardized mean differences (SMDs) for the school-level and classroom-level mediators. The largest differences concern classroom social and academic composition. Lyceum students attend classrooms with substantially higher average prior achievement in both language (SMD = 1.873) and mathematics (SMD = 1.315), as well as markedly higher average socioeconomic composition (SMD = 1.735). Sizeable differences also emerge for school climate: lyceums display more favourable disciplinary climate (SMD = 1.002), while technical schools show a more challenging behavioural climate (SMD = − 0.581).

By contrast, differences are generally smaller for instructional-quality indicators and school resources or organizational practices. Some exceptions are visible, such as instructional enjoyment (SMD = 0.537) and cultural activities (SMD = 0.468), but most indicators in these domains show substantially weaker between-track differentiation. Overall, it appears that classroom composition and school climate are the domains most likely to account for the residual lyceum–technical gap, whereas instructional quality and organizational resources have less explanatory scope unless they are strongly associated with achievement despite their more limited between-track differences.

**Table 3:** Descriptive statistics for school-level variables (unstandardized) and standardized mean differences between lyceum and technical track students

Variable	Lyceum mean (SD) (*N* = 12792)	Technical mean (SD) (*N* = 10332)	SMDs (Lyceum – technical)	*P*-value
Classroom social and academic composition				
Grade 8 language prior achievement	0.478 (0.416)	− 0.264 (0.369)	1.873	< 0.001
Grade 8 mathematics prior achievement	0.521 (0.500)	− 0.106 (0.446)	1.315	< 0.001
Classroom ESCS	0.541 (0.369)	− 0.061 (0.317)	1.735	< 0.001
School climate				
Challenging behavioural climate	1.950 (0.370)	2.182 (0.434)	− 0.581	< 0.001
Disciplinary climate	0.202 (0.405)	− 0.198 (0.393)	1.002	< 0.001
Student–teacher relations	2.739 (0.185)	2.735 (0.189)	0.020	0.1359
Instructional quality				
Teacher support	0.002 (0.265)	− 0.009 (0.260)	0.040	0.0024
Classroom management	2.768 (0.218)	2.797 (0.219)	− 0.131	< 0.001
Adaptive instruction	− 0.138 (0.274)	− 0.180 (0.264)	0.155	< 0.001
Instructional enjoyment	2.834 (0.221)	2.709 (0.246)	0.537	< 0.001
Unresponsiveness	0.248 (0.106)	0.256 (0.105)	− 0.075	< 0.001
School resources and organizational practices				
Material resources	2.173 (0.643)	2.234 (0.686)	− 0.093	< 0.001
Class size	23.026 (3.948)	22.770 (4.832)	0.059	< 0.001
School quality	0.836 (0.292)	0.840 (0.271)	− 0.014	0.2956
Cultural activities	0.595 (0.200)	0.505 (0.185)	0.468	< 0.001
Ability grouping	0.536 (0.719)	0.459 (0.552)	0.118	< 0.001
Extra hours	0.706 (0.454)	0.778 (0.414)	− 0.164	< 0.001

Values are means with standard deviations in parentheses. SMD = standardized mean difference, calculated as the difference between means of students enrolled in the Lyceum and in the Technical track divided by the pooled standard deviation; positive values indicate higher values in Lyceums. *P*-values are calculated using Welch’s two-sample t-tests

### The effect of attending the lyceum on student achievement

**Fig. 3:**
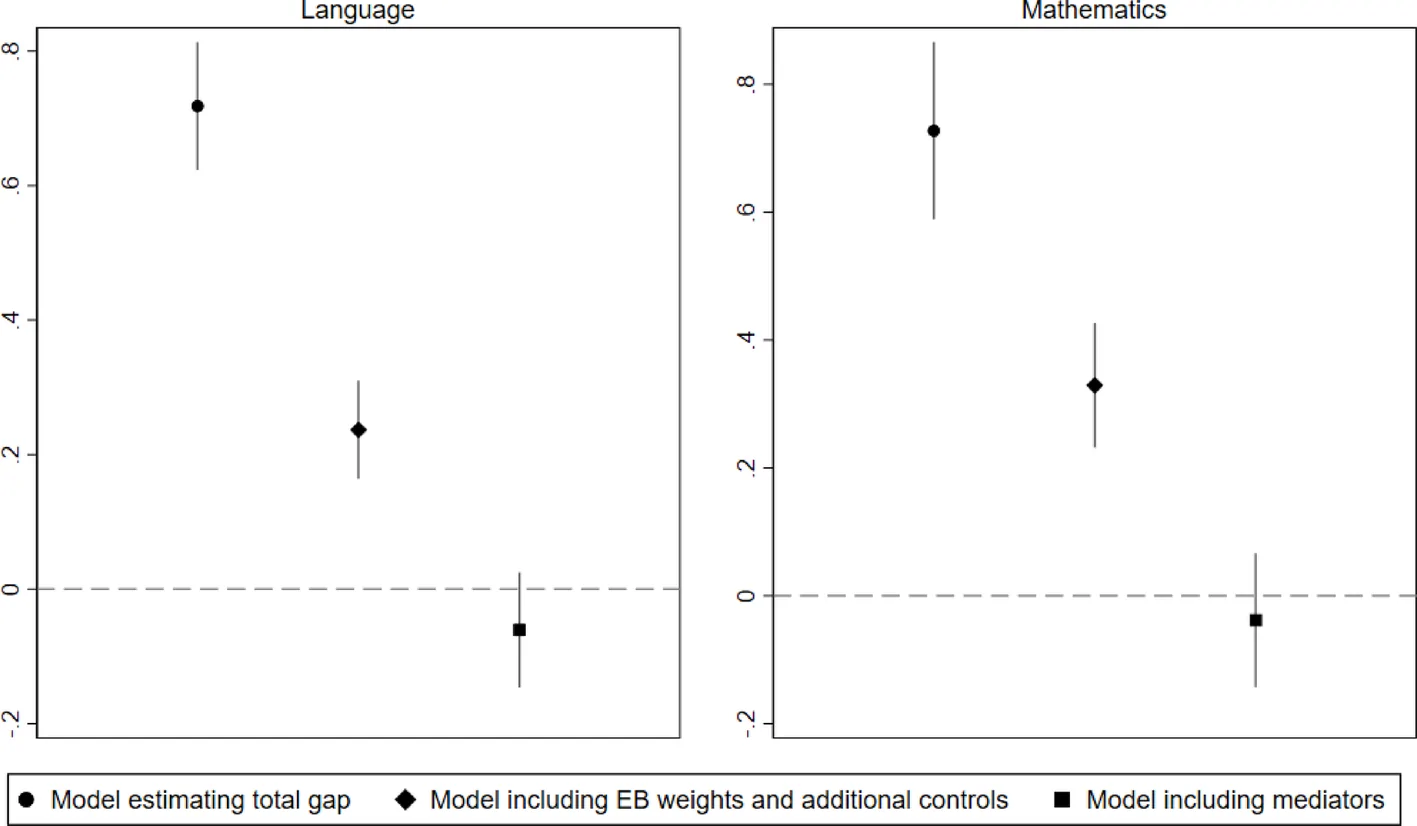
Coefficient plots of lyceum-technical schools achievement gaps in language (left) and mathematics (right): total gap, adjusted gap for selection (weighted with ebalance weights and including additional student controls), and net of school-level mediators

We estimate lyceum–technical differences in 10th-grade achievement through a sequence of increasingly demanding models designed to separate selection at entry from net track exposure and to assess the role of observed school characteristics. Model 0 (M0) reports the *unadjusted* achievement gap between students enrolled in lyceums and those in technical schools. Model 1 (M1) addresses selection into tracks by applying entropy-balancing weights and additionally controlling with regression adjustment for students’ socio-demographic characteristics and prior achievement. The contrast between M0 and M1 provides an estimate of how much of the observed lyceum advantage reflects social and institutional selectivity at entry. Model 2 (M2) further introduces school-level mediators—capturing classroom composition, school climate, instructional quality, and activities or organizational policies—to evaluate whether any *residual* gap remains once differences in observed upper-secondary school environments are taken into account. Estimated gaps across models are summarized in Fig. 3.

Results show a large raw lyceum advantage in both domains: about 0.72 SD in language and 0.73 SD in mathematics. In terms of international benchmarks, an effect of ~ 0.7 SD is substantial: on the PISA reporting scale (SD ≈ 100 points), it corresponds roughly to 70 score points. Using OECD estimates that one year of schooling is associated with roughly 30–40 PISA points (depending on country and specification), this magnitude is broadly comparable to around two school years (or more) of learning.

Once selection is addressed (M1), the estimated lyceum effect declines sharply to 0.24 SD in language and 0.33 SD in mathematics, indicating that a sizeable portion of the raw gap is attributable to pre-existing differences between students entering the two tracks. In proportional terms, selection accounts for approximately 67% of the language gap and 54% of the mathematics gap (computed as * (M0-M1)/M0*) × 100. Importantly, however, the remaining effects are still statistically significant and substantively meaningful, consistent with the idea that track attendance entails non-trivial differences in exposure over roughly two years of upper-secondary schooling. The net effect is also larger in mathematics than in language (0.329 vs. 0.237), suggesting that the academic track may be particularly consequential for domains more sensitive to curricular intensity and pacing.

In M2, after adding the school-level mediator blocks, the lyceum–technical differences further attenuate to -0.061 SD in language and − 0.038 SD in mathematics, and become not statistically distinguishable from zero at the 95% confidence level. This pattern provides initial evidence that observed school environments collectively account for much of the *residual* advantage associated with lyceum attendance after balancing on pre-treatment characteristics and adjusting for students’ sociodemographic characteristics and prior achievement. In line with our identification strategy, we interpret this as a structured accounting of the adjusted gap rather than as a causal mediation effect.

### Explaining the residual lyceum advantage: decomposition of school-environment characteristics

The KOB decompositions provide a structured assessment of how far observed school-environment characteristics account for the post-balancing lyceum–technical achievement gap. Throughout, a positive contribution indicates that the mediator helps explain the remaining lyceum advantage; a negative contribution indicates a suppressor pattern, meaning that equalising technical schools to lyceums on that characteristic would increase rather than reduce the predicted gap.

Taken jointly, the school-level mediators account for the entire residual track differential in both language and mathematics. For language, the decomposition attributes 126.2% of the adjusted lyceum–technical gap to the mediator set (*p* < 0.001; 95% CI 101.1% to 151.3%). For mathematics, the corresponding share is 111.6% (*p* < 0.001; 95% CI 94.5% to 128.6%). Contributions above 100% arise because some mediators make negative contributions, partly offsetting the positive contribution of other school-environment characteristics. Substantively, these estimates indicate that, conditional on extensive pre-treatment balancing and additional controls, observed differences in upper-secondary school environments account for the residual lyceum advantage.

**Fig. 4:**
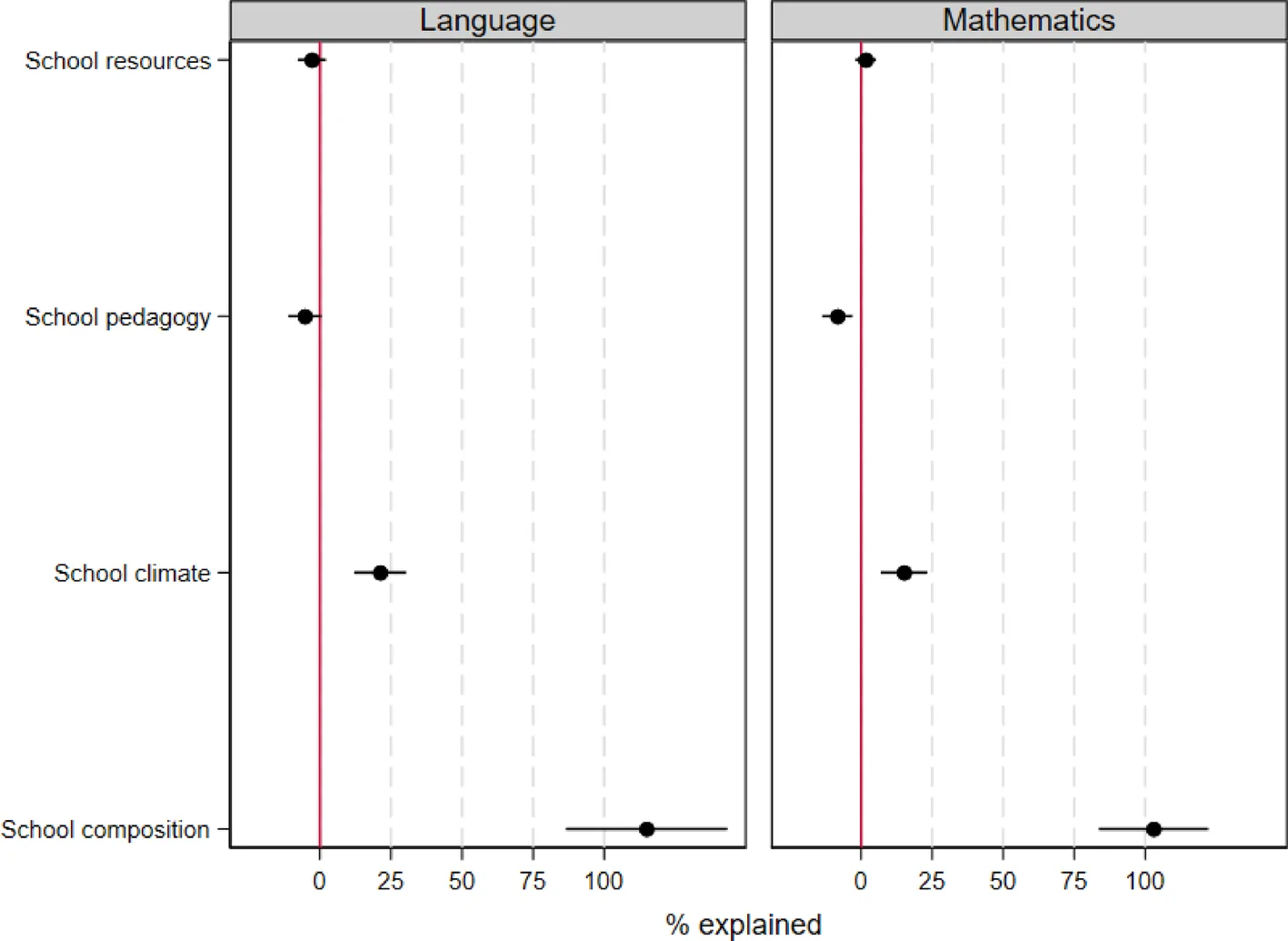
Kitagawa–Oaxaca–Blinder decomposition of the lyceums-technical schools’ net achievement gap: contribution of the four blocks of school-level mediators expressed in percentage

Figure 4 summarizes the decomposition by four broad domains: classroom composition, school climate, instructional quality, and school resources/policies. The figure shows that the explanatory component is overwhelmingly concentrated in classroom composition, with school climate playing a secondary role. By contrast, pedagogy and school resources contribute little or operate as suppressors.

**Fig. 5:**
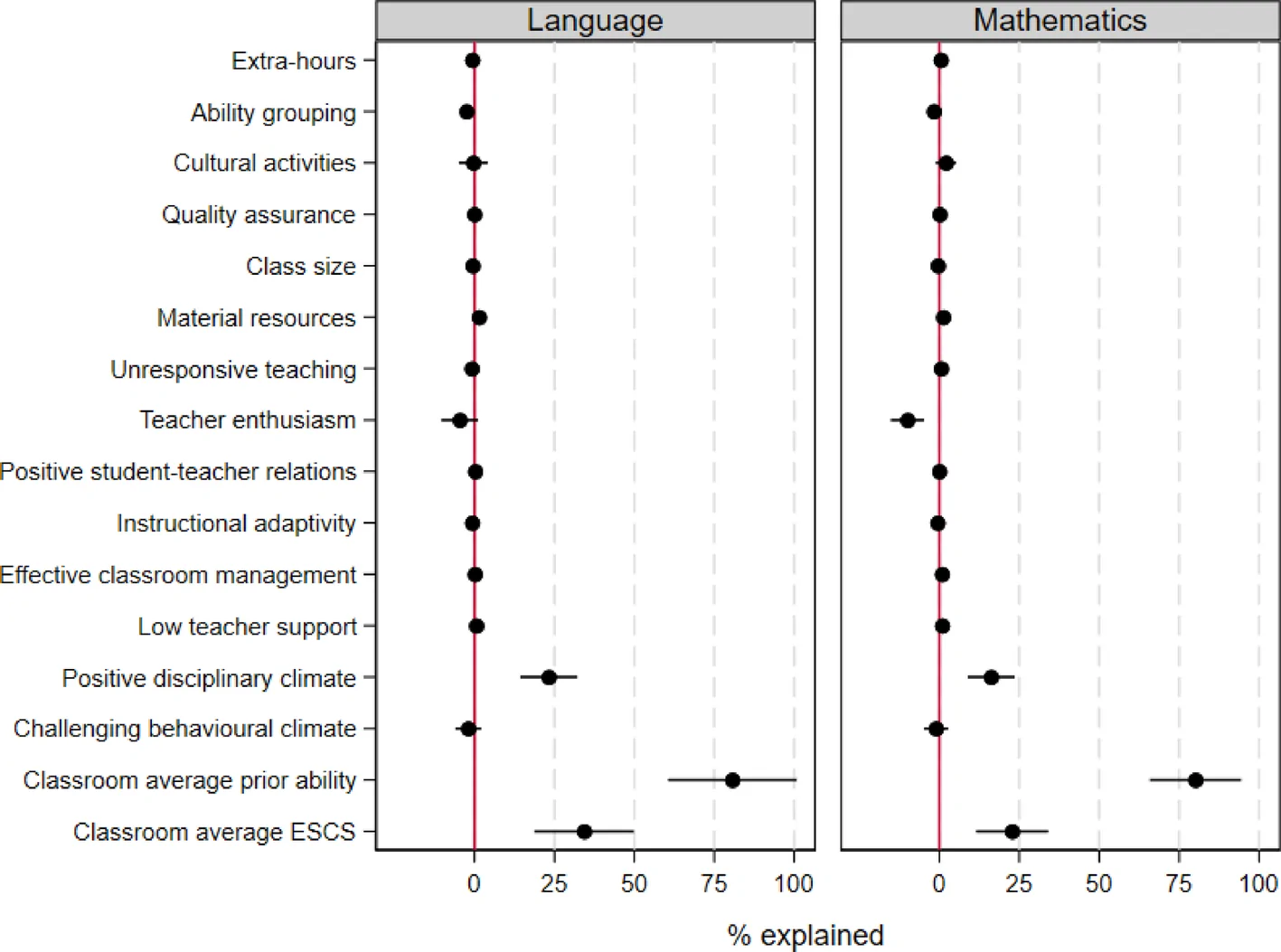
Kitagawa–Oaxaca–Blinder decomposition of the lyceums-technical schools’ net achievement gap: contribution of the detailed school-level mediators expressed in percentage

Figure 5 reports the detailed decomposition, showing the contribution of each school-environment characteristic. This more disaggregated analysis clarifies that the overall pattern is driven by a small number of mediators, while most school-level characteristics make negligible or statistically insignificant contributions once the full set of mediators is considered simultaneously.

The pattern is highly consistent across language and mathematics and confirms that the explained component is driven by a small number of mediators. The dominant factor is average classroom prior achievement, which accounts for about four-fifths of the explained gap in both domains: 80.6% in language and 80.1% in mathematics (*p* < 0.001 in both cases). Average classroom ESCS is the second most important contributor, explaining 34.3% of the language gap and 22.8% of the mathematics gap (*p* < 0.001). Among school-climate indicators, positive disciplinary climate provides the only sizeable additional contribution, accounting for 23.2% in language and 16.2% in mathematics (*p* < 0.001). These findings indicate that the residual lyceum advantage is overwhelmingly associated with the academic and socioeconomic composition of classrooms and, secondarily, with more orderly disciplinary environments.

By contrast, the remaining mediators contribute little once classroom composition and disciplinary climate are taken into account. Challenging behavioural climate is not statistically significant in either domain. Most instructional-quality indicators are also negligible: low teacher support, effective classroom management, instructional adaptivity, and positive student–teacher relations do not make clear independent contributions. Teacher enthusiasm is the main exception, but only in mathematics, where it operates as a suppressor (− 10.0%, *p* < 0.001). Within the resources and policies domain, material resources make small positive contributions in both domains — 1.4% in language and 1.2% in mathematics — while ability grouping contributes negatively, − 2.5% in language and − 1.7% in mathematics (*p* < 0.001). Overall, the detailed decomposition shows that the adjusted lyceum–technical achievement gap is explained primarily by classroom composition, especially classmates’ prior achievement, whereas instructional quality and school resources play at most a marginal independent role.

## Discussion and conclusions

This study advances research on track effects in secondary education by combining two strengths that are rarely available in the same design: population-level longitudinal achievement histories and rich pre-tracking covariates from INVALSI, and internationally standardised school-environment measures from the Italian PISA 2018 school and student questionnaires. By integrating these sources at the school level, we address a central limitation of much research on tracking: the difficulty of separating track exposure from pre-existing differences between students. At the same time, we move beyond “black-box” estimates of track gaps by examining which features of the school learning environment are associated with the residual achievement advantage of academic tracks.

Three findings emerge as particularly relevant. First, the raw achievement gap between classical/scientific lyceums and technical schools is large and similar across domains, amounting to about 0.7 standard deviations in both language and mathematics. This confirms the strong stratification of competencies by track already in Grade 10. Second, much of this gap reflects selection into tracks. Once technical-school students are reweighted to match lyceum students on prior achievement, socio-demographic characteristics, and early attitudes, the lyceum advantage shrinks markedly, although it does not disappear. This pattern is consistent with the Italian evidence showing that track enrolment reflects cumulative stratification in prior achievement and educational careers, but that track differences cannot be reduced entirely to pre-existing disparities (Triventi et al., 2021). Third, the remaining post-balancing gap is largely accounted for by observed school-environment characteristics, especially classroom academic and socioeconomic composition and, to a lesser extent, positive disciplinary climate. By contrast, instructional-quality indicators and school resources/policies contribute little once compositional and climate-related dimensions are taken into account. This limited contribution is consistent with their weaker between-track differentiation: unlike classroom composition and disciplinary climate, most indicators in these domains vary only modestly between lyceums and technical schools. Moreover, measures of teaching quality and school-related policies appear to be either negatively or only weakly associated with academic achievement, both in unconditional analyses and after adjusting for educational track attendance (lyceum versus technical track; see Appendix Figs. A1 and A2).

Our findings support a dual interpretation of the academic-track advantage. On the one hand, the large reduction in the lyceum–technical gap after entropy balancing shows that academic-track advantages are strongly rooted in selection: students entering lyceums are already more advantaged in prior achievement, social background, and school-related attitudes. On the other hand, the persistence of a non-trivial adjusted gap, and its subsequent attenuation after introducing school-environment characteristics, suggests that track placement is also associated with exposure to differentiated learning contexts. In this sense, the results are consistent with theories of tracking as a source of differential learning environments, in which institutional separation produces classrooms that differ not only in curricula and expectations, but also in peer composition, behavioural climate, and academic orientation (Gamoran & Mare, 1989; Hallinan, 1994; Maaz et al., 2008; Gamoran, 2009).

The comparison with Traini et al. (2021) is especially informative. Using German NEPS data and entropy balancing, they show that the upper-track advantage is explained mainly by average class performance and, to a lesser extent, by social-background composition, whereas migration composition and student-reported instructional quality contribute little. Our findings point in a similar direction, but extend this evidence by considering a broader set of school-environment characteristics. Despite a richer set of mediators, the central pattern remains strongly compositional: the residual lyceum advantage is explained primarily by classmates’ prior achievement and socioeconomic background, followed by positive disciplinary climate, while instructional-quality indicators and school resources play only a marginal independent role. This convergence suggests that, across different between-school tracking systems, academic-track advantages may operate less through directly observed pedagogical practices or material resources than through the compositional learning environments generated by track allocation.

Substantively, the results indicate that classroom composition is not merely a background feature of tracked systems, but a central component of the learning environment experienced by students. Average classroom prior achievement is the dominant explanatory factor in both domains, followed by classroom ESCS and positive disciplinary climate. This pattern suggests that students in academic tracks benefit from being surrounded by classmates who enter upper secondary education with stronger prior competencies and more advantaged family resources. Such compositional environments may support learning by raising the pace and level of instruction, enhancing teachers’ standards and expectations, strengthening academic norms, reducing behavioural disruption, and increasing the availability of academic information and support among peers. At the same time, the finding that disciplinary climate also contributes independently indicates that the academic-track advantage is not only a matter of peer composition, but also of the everyday order and behavioural conditions under which instruction takes place.

Several limitations qualify for this interpretation. First, entropy balancing addresses selection on observables. Although our covariate set is unusually rich and spans multiple pre-tracking stages, including prior achievement and early attitudes, unobserved determinants such as family preferences, teacher recommendations, or latent motivation may still confound the estimated post-balancing lyceum effect. Second, the decomposition analysis should be interpreted as descriptive and exploratory with respect to mechanisms. The school-level characteristics included in the decomposition may reflect institutional arrangements, resource allocation, student composition, and interactions among students and teachers. Accordingly, the explained component should not be interpreted as causal mediation or as evidence of the causal effect of individual mediators. Third, mediator measurement relies partly on PISA-based constructs observed at Grade 10 and aggregated at the school level, which introduces potential measurement error and limits the analysis of within-school heterogeneity. Finally, external validity is intentionally delimited: by focusing on classical/scientific lyceums versus technical schools, we sharpen the treatment contrast and target a substantively relevant “at-the-margin” choice in Italy, but the results should not be mechanically generalised to other lyceum types or to vocational pathways.

Despite these caveats, the findings have two broader implications. Substantively, they suggest that reducing achievement inequalities in tracked systems requires attention not only to formal track allocation, but also to the compositional and behavioural environments that tracks create. Improving disciplinary climate, strengthening classroom management, and addressing peer dynamics associated with classroom composition may help reduce achievement disparities even without immediate reforms to the formal structure of tracking. At the same time, because classroom composition itself is a product of socially and academically selective track allocation, these findings also underscore the limits of school-level interventions that leave the broader sorting process untouched.

Methodologically, the study illustrates the value of integrating national administrative panels with international survey infrastructures. INVALSI provides population-level longitudinal histories and strong leverage over pre-track selection; PISA adds information on school climate, instructional practices, and organizational features that administrative data typically lack. This integrated architecture makes it possible to estimate track contrasts with stronger adjustment for pre-tracking histories than PISA alone allows, while also examining the school-environment characteristics associated with the remaining gap.

Future research can build on this design in three directions: by exploiting quasi-exogenous variation in track placement where available; by collecting repeated longitudinal measures of school climate, classroom composition, and instructional practices; and by examining whether the residual academic-track advantage and its associated school-environment characteristics differ by baseline achievement and social background. These extensions would help clarify not only whether academic tracks generate additional learning advantages, but also for whom, when, and through which aspects of the school environment these advantages emerge.

## Supplementary Information

Below is the link to the electronic supplementary material.

Supplementary Material 1.

## Data Availability

The study is based on the secondary analysis of anonymized administrative data collected by INVALSI (Italian National Institute for the Evaluation of the Education System) and on publicly available PISA data. Access to the INVALSI data was granted through a formal agreement with the Institute and is subject to data protection regulations; these data are therefore not publicly available but can be accessed upon reasonable request and with permission from INVALSI. PISA data are openly available from the OECD. All data were fully anonymized prior to access, and all procedures complied with the General Data Protection Regulation (GDPR) and with the ethical and data protection regulations of the University of Milan.

## Footnotes

1 The Index of Economic, Social and Cultural Status (ESCS) is a composite measure of students’ socioeconomic background based on parental education, parental occupational status, and home possessions. INVALSI constructs this index following the general logic of the ESCS index originally developed in PISA, although the measure used here is derived from INVALSI data rather than directly from the PISA student questionnaire.
