# Supplementary material for: Track placement, school environments, and achievement gaps: identifying the drivers of the academic track advantage in Italy
Source: Large Scale Assess Educ. 2026 Oct 30;14(1):58. doi: 10.1186/s40536-026-00329-3 (PMC13722748; doi:10.1186/s40536-026-00329-3)
Supplement: Supplementary file 1 [file 40536_2026_329_MOESM1_ESM.docx]

# **Online Appendix**

## **Section A – Descriptive statistics and empirical checks**

Table A1 – Descriptive statistics for individual-level variables (outcomes, treatment and control variables): overall and distinguished by school track (lyceums, technical schools)

| **Variable** | **Technical** | **Lyceum** | **Total** |
| --- | --- | --- | --- |
|  |  |  |  |
| *N* | 10 332 (44.7%) | 12 792 (55.3%) | 23 124 (100%) |
|  |  |  |  |
| Italian scores (10th) | -0.259 (0.898) | 0.459 (0.916) | 0.137 (0.976) |
| Math scores (10th) | -0.073 (0.863) | 0.653 (0.995) | 0.328 (1.006) |
| Italian scores (5th) | -0.147 (0.909) | 0.408 (0.947) | 0.160 (0.970) |
| Math scores (5th) | -0.034 (0.925) | 0.414 (0.986) | 0.214 (0.985) |
| Italian scores (8th) | -0.260 (0.849) | 0.477 (0.961) | 0.148 (0.984) |
| Math scores (8th) | -0.102 (0.905) | 0.521 (1.030) | 0.242 (1.024) |
|  |  |  |  |
| Student ESCS | -0.050 (0.855) | 0.553 (0.953) | 0.283 (0.959) |
|  |  |  |  |
| Student sex |  |  |  |
| Male | 7 117 (68.8%) | 6 614 (51.7%) | 13 731 (59.38%) |
| Female | 3 215 (31.1%) | 6 178 (48.3%) | 9 393 (40.6%) |
|  |  |  |  |
| Migration background |  |  |  |
| Natives | 8 715 (84.3%) | 11 476 (89.7%) | 20 191 (87.3%) |
| Mixed | 686 (6.6%) | 689 (5.39%) | 1 375 (5.95%) |
| 2GEN | 618 (5.98%) | 463 (3.6%) | 1 081 (4.67%) |
| 1GEN | 313 (3%) | 164 (1.28%) | 477 (2.06%) |
|  |  |  |  |
| Geographical area (5th) |  |  |  |
| North-West | 1 729 (16.7%) | 2 323 (18.16%) | 4 052 (17.5%) |
| North-East | 2 543 (24.6%) | 2 063 (16.1%) | 4 606 (19.9%) |
| Center | 3 061 (29.6%) | 3 022 (23.6%) | 6 083 (26.3%) |
| South | 1 699 (16.4%) | 2 929 (22.9%) | 4 628 (20.01%) |
| Isles | 1 300 (12.58%) | 2 455 (19.19%) | 3 755 (16.24%) |
|  |  |  |  |
| Geographical area (8th) |  |  |  |
| North-West | 1 731 (16.75%) | 2 310 (18.06%) | 4 041 (17.48%) |
| North-East | 2 548 (24.66%) | 2 067 (16.16%) | 4 615 (19.96%) |
| Center | 3 072 (29.7%) | 3 026 (23.66%) | 6 098 (26.37%) |
| South | 1 682 (16.28%) | 2 933 (22.93%) | 4 615 (19.96%) |
| Isles | 1 299 (12.57%) | 2 456 (19.2%) | 3 755 (16.24%) |
|  |  |  |  |
| Number of siblings | 1.139 (0.803) | 1.103 (0.765) | 1.119 (0.782) |
|  |  |  |  |
| Reading appreciation (5th) | 2.989 (1.577) | 3.282 (1.470) | 3.151 (1.526) |
| Math appreciation (5th) | 0.048 (0.974) | 0.225 (0.874) | 0.146 (0.925) |
| Academic diligence (5th) | 3.293 (0.800) | 3.434 (0.730) | 3.371 (0.766) |
| External motivation (5th) | 8.011 (3.111) | 6.919 (2.778) | 7.407 (2.981) |
| Test anxiety (5th) | 6.634 (2.304) | 6.220 (2.143) | 6.405 (2.226) |
|  |  |  |  |
| Student condition |  |  |  |
| Regular | 10 116 (97.9%) | 12 430 (97.17%) | 22 546 (97.5%) |
| Early entrant | 52 (0.5%) | 281 (2.2%) | 333 (1.44%) |
| Late entrant | 164 (1.59%) | 81 (0.63%) | 245 (1.06%) |

*Note.* Mean values and standard deviations in parentheses for continuous variables, number of observations and percentages in parentheses for categorical variables.

Table A2 – Descriptive statistics for school-level variables (unstandardized): overall and distinguished by school track (lyceums, technical schools)

| **Variable** | **Technical** | **Lyceum** | **Total** |
| --- | --- | --- | --- |
|  |  |  |  |
| *N* | 10 332 (44.7%) | 12 792 (55.3%) | 23 124 (100%) |
| Average classroom ESCS | -0.061 (0.317) | 0.541 (0.369) | 0.272 (0.458) |
| Classroom average prior language achievement | -0.263 (0.368) | 0.477 (0.416) | 0.146 (0.540) |
| Classroom average prior mathematics achievement | -0.106 (0.446) | 0.521 (0.500) | 0.241 (0.570) |
| Challenging behavioural climate | 2.182 (0.434) | 1.950 (0.369) | 2.054 (0.416) |
| Positive disciplinary climate | -0.198 (0.393) | 0.202 (0.405) | 0.023 (0.446) |
| Low teacher support | -0.008 (0.259) | 0.0019 (0.265) | -0.003 (0.263) |
| Effective classroom management | 2.796 (0.219) | 2.768 (0.218) | 2.781 (0.219) |
| Instructional adaptivity | -0.179 (0.264) | -0.138 (0.273) | -0.157 (0.270) |
| Positive student-teacher relations | 2.734 (0.189) | 2.738 (0.184) | 2.737 (0.186) |
| Teacher enthusiasm | 2.709 (0.245) | 2.833 (0.221) | 2.778 (0.240) |
| Teacher unresponsiveness | 0.255 (0.105) | 0.247 (0.106) | 0.251 (0.106) |
| Material resources | 2.234 (0.686) | 2.173 (0.642) | 2.200 (0.663) |
| Classroom size | 22.769 (4.831) | 23.026 (3.948) | 22.911 (4.366) |
| Quality assurance | 0.839 (0.271) | 0.836 (0.292) | 0.837 (0.283) |
| School cultural activities | 0.504 (0.184) | 0.595 (0.200) | 0.555 (0.198) |
| Ability grouping | 0.459 (0.551) | 0.535 (0.718) | 0.501 (0.651) |
| Extra instructional hours | 0.777 (0.413) | 0.705 (0.454) | 0.737 (0.438) |

*Note.* Mean values and standard deviations in parentheses for continuous variables.

Table A3 – Comparison between students included in the PISA-INVALSI matched sample and excluded Grade 10 INVALSI students

| **Variable** | **Excluded students**  ***N* = 287 566**  **Mean (SD)** | **Included students**  ***N* = 40 568**  **Mean (SD)** | **Standardized Mean Difference** |
| --- | --- | --- | --- |
| Italian achievement (Grade 10) | 0.000 (1.000) | -0.000 (1.003) | -0.000 |
| Mathematics achievement (Grade 10) | -0.005 (0.998) | 0.037 (1.014) | 0.042 |
| Student ESCS | 0.159 (0.980) | 0.183 (0.965) | 0.024 |
| Relative birth year | 0.083 (0.337) | 0.066 (0.317) | -0.052 |
| Female (*ref*. Male) | 0.520 (0.500) | 0.517 (0.500) | -0.007 |

Table A4 – Comparison between schools included and excluded from the PISA-INVALSI matched sample

| **Variable** | **Excluded schools**  ***N* = 3 347**  **Mean (SD)** | **Included schools**  ***N* = 351**  **Mean (SD)** | **Standardized Mean Difference** |
| --- | --- | --- | --- |
| Average Italian achievement | -0.163 (0.618) | -0.178 (0.581) | -0.025 |
| Average mathematics achievement | -0.144 (0.640) | -0.144 (0.628) | 0.000 |
| Average school ESCS | 0.165 (0.558) | 0.101 (0.409) | -0.131 |
| School size | 85.9 (69.2) | 115.6 (65.6) | 0.440 |
| Share of female students | 0.501 (0.248) | 0.508 (0.205) | 0.032 |
| Share of older students | 0.018 (0.035) | 0.018 (0.021) | 0.012 |
| Share of on-time students | 0.879 (0.122) | 0.902 (0.072) | 0.223 |
| Share younger students | 0.103 (0.120) | 0.080 (0.073) | -0.229 |

*Notes for Tables A3 and A4.* Student-level comparisons are based on the full Grade 10 INVALSI population. School-level comparisons are based on all Grade 10 schools observed in INVALSI. Included observations refer to the analytical PISA-INVALSI matched sample used in the main analyses. Standardized mean differences (SMDs) quantify differences in observable characteristics between included and excluded observations.

Figure A1 – Conditional (on educational track attendance) and unconditional associations between school-level mediators and Grade 10 language achievement

Figure A2 – Conditional (on educational track attendance) and unconditional associations between school-level mediators and Grade 10 mathematics achievement

## **Section B – Tables behind graphs in the manuscript**

Table B1 - Balancing table of pre-tracking individual-level characteristics across lyceums versus technical schools: before and after ebalancing weights.

**Entropy Balancing – Descriptive Statistics**

**Treated units (Lyceum-track students):** 14 202 (total weights = 14 202) 
**Control units (Technical-track students):** 11 106 (total weights = 14 202)

The total weights (14 202) are used to statistically replicate the characteristics of the control group

**Before weighting**

| **Variable** | **Treated units** | | | **Control units** | | |
| --- | --- | --- | --- | --- | --- | --- |
|  | *Mean* | *Variance* | *Skewness* | *Mean* | *Variance* | *Skewness* |
| Ita test scores (G5) | 0.41 | 0.89 | −0.58 | −0.16 | 0.84 | −0.40 |
| Math test scores (G5) | 0.42 | 0.96 | −0.33 | −0.04 | 0.87 | −0.16 |
| Ita test scores (G8) | 0.49 | 0.92 | 0.05 | −0.27 | 0.73 | 0.07 |
| Math test scores (G8) | 0.52 | 1.06 | −0.18 | −0.11 | 0.82 | 0.12 |
| ESCS | 0.56 | 0.90 | −0.06 | −0.06 | 0.74 | 0.12 |
| Sex: Female (ref. Male) | 0.48 | 0.25 | 0.06 | 0.31 | 0.21 | 0.83 |
| Migration background: Mixed (ref. Natives) | 0.05 | 0.05 | 3.95 | 0.06 | 0.06 | 3.55 |
| Migration background: IIGEN (ref. Natives) | 0.04 | 0.03 | 5.02 | 0.06 | 0.05 | 3.80 |
| Migration background: IGEN (ref. Natives) | 0.01 | 0.01 | 8.52 | 0.03 | 0.03 | 5.53 |
| Geo area of residence (G5): North-East (ref. North-West) | 0.18 | 0.15 | 1.70 | 0.26 | 0.19 | 1.11 |
| Geo area of residence (G5): Center (ref. North-West) | 0.24 | 0.18 | 1.21 | 0.28 | 0.20 | 0.96 |
| Geo area of residence (G5): South (ref. North-West) | 0.23 | 0.18 | 1.31 | 0.18 | 0.15 | 1.65 |
| Geo area of residence (G5): Isles (ref. North-West) | 0.18 | 0.15 | 1.65 | 0.12 | 0.11 | 2.34 |
| Geo area of residence (G8): North-East (ref. North-West) | 0.18 | 0.15 | 1.69 | 0.26 | 0.19 | 1.11 |
| Geo area of residence (G8): Center (ref. North-West) | 0.24 | 0.18 | 1.21 | 0.29 | 0.20 | 0.95 |
| Geo area of residence (G8): South (ref. North-West) | 0.23 | 0.18 | 1.31 | 0.18 | 0.15 | 1.67 |
| Geo area of residence (G8): Isles (ref. North-West) | 0.18 | 0.15 | 1.65 | 0.12 | 0.11 | 2.34 |
| Number of siblings (G5) | 1.11 | 0.59 | 0.95 | 1.14 | 0.64 | 0.98 |
| Index measuring reading appreciation (G5) | 0.01 | 0.89 | −0.60 | −0.18 | 1.04 | −0.37 |
| Index measuring mathematics appreciation (G5) | 0.23 | 0.77 | −0.91 | 0.05 | 0.95 | −0.67 |
| Index measuring academic diligence (G5) | 0.11 | 0.85 | −1.28 | −0.07 | 1.04 | −1.01 |
| Index measuring external motivation (G5) | −0.18 | 0.82 | 0.93 | 0.19 | 1.04 | 0.50 |
| Index measuring test anxiety (G5) | −0.20 | 0.86 | 0.41 | −0.02 | 1.00 | 0.24 |
| Student status (G8): Early entrant (ref. On-time entrant) | 0.02 | 0.02 | 6.44 | 0.01 | 0.01 | 12.96 |
| Student status (G8): Late entrant (ref. On-time entrant) | 0.01 | 0.01 | 12.30 | 0.02 | 0.02 | 7.75 |

**After weighting**

| **Variable** | **Treated units** | | | **Control units** | | |
| --- | --- | --- | --- | --- | --- | --- |
|  | *Mean* | *Variance* | *Skewness* | *Mean* | *Variance* | *Skewness* |
| Ita test scores (G5) | 0.41 | 0.89 | −0.58 | 0.41 | 0.89 | −0.58 |
| Math test scores (G5) | 0.42 | 0.96 | −0.33 | 0.42 | 0.96 | −0.33 |
| Ita test scores (G8) | 0.49 | 0.92 | 0.05 | 0.49 | 0.92 | 0.05 |
| Math test scores (G8) | 0.52 | 1.06 | −0.18 | 0.52 | 1.06 | −0.18 |
| ESCS | 0.56 | 0.90 | −0.06 | 0.56 | 0.90 | −0.06 |
| Sex: Female (*ref*. Male) | 0.48 | 0.25 | 0.06 | 0.48 | 0.25 | 0.06 |
| Migration background: Mixed (*ref*. Natives) | 0.05 | 0.05 | 3.95 | 0.05 | 0.05 | 3.95 |
| Migration background: IIGEN (*ref*. Natives) | 0.04 | 0.03 | 5.02 | 0.04 | 0.03 | 5.02 |
| Migration background: IGEN (*ref*. Natives) | 0.01 | 0.01 | 8.52 | 0.01 | 0.01 | 8.52 |
| Geo area of residence (G5): North-East (*ref*. North-West) | 0.18 | 0.15 | 1.70 | 0.18 | 0.15 | 1.70 |
| Geo area of residence (G5): Center (*ref*. North-West) | 0.24 | 0.18 | 1.21 | 0.24 | 0.18 | 1.21 |
| Geo area of residence (G5): South (*ref*. North-West) | 0.23 | 0.18 | 1.31 | 0.23 | 0.18 | 1.31 |
| Geo area of residence (G5): Isles (*ref*. North-West) | 0.18 | 0.15 | 1.65 | 0.18 | 0.15 | 1.65 |
| Geo area of residence (G8): North-East (*ref*. North-West) | 0.18 | 0.15 | 1.69 | 0.18 | 0.15 | 1.69 |
| Geo area of residence (G8): Center (*ref*. North-West) | 0.24 | 0.18 | 1.21 | 0.24 | 0.18 | 1.21 |
| Geo area of residence (G8): South (*ref*. North-West) | 0.23 | 0.18 | 1.31 | 0.23 | 0.18 | 1.31 |
| Geo area of residence (G8): Isles (*ref*. North-West) | 0.18 | 0.15 | 1.65 | 0.18 | 0.15 | 1.65 |
| Number of siblings (G5) | 1.11 | 0.59 | 0.95 | 1.11 | 0.59 | 0.95 |
| Index measuring reading appreciation (G5) | 0.01 | 0.89 | −0.60 | 0.01 | 0.89 | −0.60 |
| Index measuring mathematics appreciation (G5) | 0.23 | 0.77 | −0.91 | 0.23 | 0.77 | −0.91 |
| Index measuring academic diligence (G5) | 0.11 | 0.85 | −1.28 | 0.11 | 0.85 | −1.28 |
| Index measuring external motivation (G5) | −0.18 | 0.82 | 0.93 | −0.18 | 0.82 | 0.93 |
| Index measuring test anxiety (G5) | −0.20 | 0.86 | 0.41 | −0.20 | 0.86 | 0.41 |
| Student status (G8): Early entrant (*ref*. On-time entrant) | 0.02 | 0.02 | 6.44 | 0.02 | 0.02 | 6.44 |
| Student status (G8): Late entrant (*ref*. On-time entrant) | 0.01 | 0.01 | 12.30 | 0.01 | 0.01 | 12.30 |

Table B2 - Lyceum-technical schools achievement gaps in language and mathematics: total gap (M0), adjusted gap for selection and including additional controls (M1: weighted with *ebalance* weights and controlling for prior achievement, in Grade 8, and student socio-demographic characteristics), and net of school-level mediators (M2)

**Language achievement in Grade 10**

| **Variable** | **M0** | **M1** | **M2** |
| --- | --- | --- | --- |
| **Track** (*ref*. Technical) |  |  |  |
| Lyceum | 0.718*** (0.048) | 0.237*** (0.037) | -0.061 (0.043) |
| **Student controls** |  |  |  |
| Student ESCS |  | 0.066*** (0.011) | 0.026** (0.010) |
| Female (*ref*. Male) |  | 0.061** (0.025) | 0.034 (0.021) |
| Mixed migration background (*ref*. Non-migrant) |  | 0.013 (0.035) | 0.019 (0.033) |
| Second-generation immigrant (*ref*. Non-migrant) |  | 0.024 (0.037) | -0.030 (0.036) |
| First-generation immigrant (*ref*. Non-migrant) |  | 0.053 (0.060) | -0.001 (0.065) |
| Prior reading achievement (Grade 8) |  | 0.511*** (0.015) | 0.469*** (0.014) |
| **School composition** |  |  |  |
| Average classroom ESCS |  |  | 0.151*** (0.039) |
| Classroom average prior language ability |  |  | 0.272*** (0.036) |
| **School climate** |  |  |  |
| Challenging behavioural climate (std) |  |  | 0.010 (0.020) |
| Positive disciplinary climate (std) |  |  | 0.064*** (0.022) |
| **Pedagogy** |  |  |  |
| Low teacher support (std) |  |  | 0.028 (0.026) |
| Effective classroom management (std) |  |  | -0.003 (0.024) |
| Instructional adaptivity |  |  | -0.024 (0.027) |
| Positive student-teacher relations (std) |  |  | 0.018 (0.025) |
| Teacher enthusiasm (std) |  |  | -0.021 (0.021) |
| Unresponsive teaching |  |  | 0.181 (0.188) |
| **School resources** |  |  |  |
| Material resources (std) |  |  | -0.033** (0.016) |
| Classroom size |  |  | 0.010*** (0.002) |
| Quality assurance (std) |  |  | -0.002 (0.016) |
| School cultural activities (std) |  |  | -0.002 (0.016) |
| Ability grouping (std) |  |  | -0.051*** (0.016) |
| Extra instructional hours (std) |  |  | 0.012 (0.014) |
| Constant | -0.259*** (0.039) | -0.090*** (0.034) | -0.229*** (0.076) |
| N | 23 124 | 23 124 | 23 124 |

*Note.* Standard errors in parentheses. * p<0.10, ** p<0.05, *** p<0.01.

**Mathematics achievement in Grade 10**

| ***Variable*** | **M0** | **M1** | **M2** |
| --- | --- | --- | --- |
| **Track** (*ref*. Technical) |  |  |  |
| Lyceum | 0.727*** (0.070) | 0.329*** (0.050) | -0.038 (0.053) |
| **Student controls** |  |  |  |
| Student ESCS |  | 0.067*** (0.014) | 0.021* (0.012) |
| Female (*ref*. Male) |  | -0.064** (0.027) | -0.083*** (0.023) |
| Mixed migration background (*ref*. Non-migrant) |  | 0.042 (0.034) | 0.047 (0.033) |
| Second-generation immigrant (*ref*. Non-migrant) |  | 0.080** (0.036) | -0.028 (0.034) |
| First-generation immigrant (*ref*. Non-migrant) |  | 0.040 (0.050) | -0.052 (0.058) |
| Prior reading achievement (Grade 8) |  | 0.578*** (0.019) | 0.492*** (0.017) |
| **School composition** |  |  |  |
| Average classroom ESCS |  |  | 0.143*** (0.049) |
| Classroom average prior mathematics ability |  |  | 0.436*** (0.042) |
| **School climate** |  |  |  |
| Challenging behavioural climate (std) |  |  | 0.007 (0.022) |
| Positive disciplinary climate (std) |  |  | 0.063** (0.029) |
| **Pedagogy** |  |  |  |
| Low teacher support (std) |  |  | 0.065** (0.031) |
| Effective classroom management (std) |  |  | -0.030 (0.031) |
| Instructional adaptivity |  |  | -0.025 (0.031) |
| Positive student-teacher relations (std) |  |  | -0.005 (0.029) |
| Teacher enthusiasm (std) |  |  | -0.066* (0.033) |
| Unresponsive teaching |  |  | -0.166 (0.203) |
| **School resources** |  |  |  |
| Material resources (std) |  |  | -0.040** (0.018) |
| Classroom size |  |  | 0.010*** (0.004) |
| Quality assurance (std) |  |  | 0.007 (0.017) |
| School cultural activities (std) |  |  | 0.013 (0.021) |
| Ability grouping (std) |  |  | -0.050*** (0.018) |
| Extra instructional hours (std) |  |  | -0.011 (0.016) |
| Constant | -0.074 (0.051) | 0.011 (0.037) | -0.052 (0.098) |
| N | 23 124 | 23 124 | 23 124 |

*Note.* Standard errors in parentheses. * p<0.10, ** p<0.05, *** p<0.01.

Table B3 - Kitagawa–Oaxaca–Blinder decomposition of the lyceums-technical schools net achievement gap (language competencies): contribution of the four blocks of school-level mediators

| **Component** | **Coefficient** | **Standard Error** | ***Z*** | **p-value** | **95% Confidence Interval** |
| --- | --- | --- | --- | --- | --- |
| Lyceum-track students (*g1*) | 0.459 | 0.008 | 56.60 | 0.000 | [0.443 ; 0.475] |
| Technical-track students (*g2*) | 0.227 | 0.022 | 10.43 | 0.000 | [0.184 ; 0.269] |
| Difference  (*g1 – g2*) | 0.231 | 0.023 | 9.97 | 0.000 | [0.186 ; 0.277] |
| Explained component | 0.292 | 0.025 | 11.57 | 0.000 | [0.242 ; 0.341] |
| Unexplained component | -0.060 | 0.026 | -2.31 | 0.021 | [-0.112 ; -0.009] |
| **Composition (explained)** | 0.2662 | 0.0214 | 12.40 | 0.000 | [0.224 ; 0.308] |
| **Climate (explained)** | 0.0491 | 0.0102 | 4.81 | 0.000 | [0.029 ; 0.069] |
| **Pedagogy (explained)** | -0.0122 | 0.0068 | -1.79 | 0.073 | [-0.025 ; 0.001] |
| **Resources (explained)** | -0.0065 | 0.0058 | -1.13 | 0.260 | [-0.017 ; 0.005] |
| Student ESCS | -0.0001 | 0.0006 | -0.24 | 0.808 | [-0.001 ; 0.001] |
| Female (*ref*. Males) | -0.0002 | 0.0004 | -0.47 | 0.636 | [-0.001 ; 0.0006] |
| Migration (Mixed, *ref*. Non-migrants) | -0.0000 | 0.0001 | -0.35 | 0.725 | [-0.0002 ; 0.0002] |
| Migration (2^nd^ generation, *ref*. Non-migrants) | 0.0000 | 0.0001 | 0.29 | 0.771 | [-0.0002 ; 0.0002] |
| Migration (1^st^ generation, *ref*. Non-migrants) | -0.0000 | 0.0005 | 0.02 | 0.987 | [-0.0001 ; 0.0001] |
| Language achievement in Grade 8 | -0.0039 | 0.0127 | -0.31 | 0.756 | [-0.028 ; 0.021] |
| **Composition (unexplained)** | -0.0260 | 0.0171 | -1.52 | 0.129 | [-0.059 ; 0.007] |
| **Climate (unexplained)** | 0.0034 | 0.0032 | 1.07 | 0.283 | [-0.003 ; 0.009] |
| **Pedagogy (unexplained)** | -0.0037 | 0.0459 | -0.08 | 0.934 | [-0.093 ; 0.086] |
| **Resources (unexplained)** | -0.0028 | 0.0830 | -0.03 | 0.972 | **[-0.165 ; 0.159]** |
| Student ESCS | 0.0006 | 0.0115 | 0.06 | 0.952 | [-0.021 ; 0.023] |
| Female (*ref*. Males) | -0.0114 | 0.0169 | -0.67 | 0.502 | [-0.044 ; 0.022] |
| Migration (Mixed, *ref*. Non-migrants) | -0.0019 | 0.0033 | -0.58 | 0.564 | [-0.008 ; 0.001] |
| Migration (2^nd^ generation, *ref*. Non-migrants) | -0.0033 | 0.0024 | -1.37 | 0.172 | [-0.008 ; 0.001] |
| Migration (1^st^ generation, *ref*. Non-migrants) | -0.0033 | 0.0024 | -1.37 | 0.172 | [-0.0080 ; 0.0014] |
| Language achievement in Grade 8 | -0.003 | 0.0016 | -1.99 | 0.046 | [-0.067 ; -0.028] |
| Constant | 0.035 | 0.099 | 0.36 | 0.718 | [-0.158 ; 0.230] |

*Note.* Blinder-Oaxaca decomposition with coefficients from a pooled linear model including a group dummy.
Group 2: Technical track students (N = 10 332); Group 1: Lyceum track students (N = 12 792); total N = 23 124.
Significance levels: * p<0.10, ** p<0.05, *** p<0.01.

Percentage contribution of blocks of mediators to the track-related language gap

| **Block** | **% explained** | **p-value** |
| --- | --- | --- |
| Composition | 114.92 | 0.000 |
| School climate | 21.23 | 0.000 |
| Pedagogy | -5.28 | 0.075 |
| School resources | -2.82 | 0.264 |

*Note.* **Composition** includes average classroom socioeconomic composition (ESCS) and classroom average prior ability. **School climate** includes behavioural and disciplinary climate. **Pedagogy** includes teacher support, classroom management, adaptivity, student-teacher relations, instructional enjoyment, and unresponsive teachers. **School resources** includes material resources, class size, educational resources, cultural activities, ability grouping, and extra instructional hours. Percentages are calculated relative to the overall achievement gap and therefore may not sum to 100% because some blocks make negative contributions.

Table B4 - Kitagawa–Oaxaca–Blinder decomposition of the lyceums-technical schools net achievement gap (mathematics competencies): contribution of the four blocks of school-level mediators

| **Component** | **Coefficient** | **Standard Error** | ***Z*** | **P-value** | **95% Confidence Interval** |
| --- | --- | --- | --- | --- | --- |
| Lyceum-track students (*g1*) | 0.6533 | 0.0088 | 74.23 | 0.000 | [0.636 ; 0.670] |
| Technical-track students (*g2*) | 0.323 | 0.024 | 13.53 | 0.000 | [0.277 ; 0.371] |
| Difference (*g1 – g2*) | 0.329 | 0.025 | 12.91 | 0.000 | [0.279 ; 0.379] |
| Explained component | 0.367 | 0.028 | 13.22 | 0.000 | [0.313 ; 0.422] |
| Unexplained component | -0.038 | 0.027 | -1.40 | 0.161 | [-0.091 ; 0.015] |
| **Composition (explained)** | 0.339 | 0.0224 | 15.10 | 0.000 | [0.295 ; 0.383] |
| **Climate (explained)** | 0.05 | 0.0137 | 3.62 | 0.000 | [0.023 ; 0.077] |
| **Pedagogy (explained)** | -0.027 | 0.0087 | -3.13 | 0.002 | [-0.044 ; -0.010] |
| **Resources (explained)** | 0.005 | 0.0061 | 0.89 | 0.376 | [-0.006 ; 0.017] |
| Student ESCS | -0.0001 | 0.0005 | -0.24 | 0.809 | [-0.0012 ; 0.0009] |
| Female (*ref*. Males) | 0.0004 | 0.0010 | 0.48 | 0.628 | [-0.001 ; 0.002] |
| Migration (Mixed, *ref*. Non-migrants) | -0.0001 | 0.0002 | -0.42 | 0.676 | [-0.0005 ; 0.0003] |
| Migration (2^nd^ generation, *ref*. Non-migrants) | 0.0000 | 0.0001 | 0.29 | 0.772 | [-0.0002 ; 0.0002] |
| Migration (1^st^ generation, *ref*. Non-migrants) | 0.0000 | 0.0001 | 0.40 | 0.692 | [-0.0001 ; 0.0002] |
| Mathematics achievement in Grade 8 | 0.0004 | 0.0136 | 0.03 | 0.974 | [-0.026 ; 0.027] |
| **Composition (unexplained)** | -0.018 | 0.0205 | -0.89 | 0.376 | [-0.058 ;  0.022] |
| **Climate (unexplained)** | 0.009 | 0.0036 | 2.66 | 0.008 | [0.0024 ; 0.016] |
| **Pedagogy (unexplained)** | -0.027 | 0.0530 | -0.51 | 0.607 | [-0.131 ; 0.076] |
| **Resources (unexplained)** | -0.219 | 0.0795 | -2.76 | 0.006 | [-0.375 ; 0.063] |
| Student ESCS | -0.001 | 0.0129 | -0.08 | 0.937 | [-0.026 ;  0.024] |
| Female (*ref*. Males) | -0.024 | 0.0188 | -1.28 | 0.199 | [-0.061 ; 0.012] |
| Migration (Mixed, *ref*. Non-migrants) | -0.001 | 0.003 | -0.54 | 0.592 | [-0.007 ; 0.004] |
| Migration (2^nd^ generation, *ref*. Non-migrants) | -0.0019 | 0.0023 | -0.83 | 0.404 | [-0.006 ; 0.002] |
| Migration (1^st^ generation, *ref*. Non-migrants) | -0.002 | 0.0013 | -1.72 | 0.085 | [-0.005 ; 0.0003] |
| Mathematics achievement in Grade 8 | -0.033 | 0.0156 | -2.11 | 0.035 | [-0.063 ; 0.002] |
| Constant | 0.281 | 0.0954 | 2.94 | 0.003 | [0.093 ; 0.468] |

*Note.* Blinder-Oaxaca decomposition with coefficients from a pooled linear model including a group dummy.
Group 2: Technical track students (N = 10 332); Group 1: Lyceum track students (N = 12 792); total N = 23 124.
Significance levels: * p<0.10, ** p<0.05, *** p<0.01.

Percentage contribution of blocks of mediators to the explained mathematics gap

| **Block** | **% explained** | **p-value** |
| --- | --- | --- |
| Composition | 102.89 | 0.000 |
| School climate | 15.13 | 0.000 |
| Pedagogy | -8.30 | 0.002 |
| School resources | 1.63 | 0.376 |

*Note.* **Composition** includes average classroom socioeconomic composition (ESCS) and classroom average prior ability. **School climate** includes behavioural and disciplinary climate. **Pedagogy** includes teacher support, classroom management, adaptivity, student-teacher relations, instructional enjoyment, and unresponsive teachers. **School resources** includes material resources, class size, educational resources, cultural activities, ability grouping, and extra instructional hours. Percentages are calculated relative to the overall mathematics achievement gap and therefore may not sum to 100% because some blocks contribute negatively.

Table B5 – Kitagawa-Oaxaca-Blinder decomposition of the lyceums-technical schools net achievement gap (language competencies): contribution of the detailed school-level mediators

| **Component** | **Coefficient** | **Standard Error** | ***Z*** | **P-value** | **95% Confidence Interval** |
| --- | --- | --- | --- | --- | --- |
| Lyceum-track students (*g1*) | 0.458 | 0.0081 | 56.59 | 0.000 | [0.442 ; 0.474] |
| Technical-track students (*g2*) | 0.227 | 0.0217 | 10.43 | 0.000 | [0.184 ; 0.269] |
| Difference (*g1 – g2*) | 0.231 | 0.0232 | 9.97 | 0.000 | [0.186 ; 0.277] |
| Explained component | 0.292 | 0.0252 | 11.57 | 0.000 | [0.242 ; 0.342] |
| Unexplained component | -0.060 | 0.0262 | -2.31 | 0.021 | [-0.112 ; -0.009] |
| **Average classroom ESCS – Explained component** | 0.079 | 0.0162 | 4.87 | 0.000 | [0.047 ; 0.111] |
| **Classroom average prior language achievement – Explained component** | 0.186 | 0.0162 | 11.50 | 0.000 | [0.155 ; 0.218] |
| **Challenging behavioural climate (std) – Explained component** | -0.004 | 0.0047 | -0.97 | 0.334 | [-0.014 ; 0.004] |
| **Positive disciplinary climate (std) – Explained component** | 0.053 | 0.0096 | 5.60 | 0.000 | [0.034 ; 0.072] |
| **Low teacher support (std) – Explained component** | 0.001 | 0.0009 | 1.47 | 0.141 | [-0.0004 ; 0.0029] |
| **Effective classroom management (std) – Explained component** | 0.0003 | 0.0011 | 0.27 | 0.788 | [-0.002 ; 0.002] |
| **Instructional adaptivity – Explained component** | -0.0017 | 0.0011 | -1.48 | 0.140 | [-0.004 ; 0.0005] |
| **Positive student-teacher relations (std) – Explained component** | 0.0005 | 0.0005 | 0.91 | 0.362 | [-0.0006 ; 0.0016] |
| **Teacher enthusiasm (std) – Explained component** | -0.010 | 0.0067 | -1.58 | 0.114 | [-0.024 ; 0.002] |
| **Unresponsive teaching – Explained component** | -0.002 | 0.0010 | -1.88 | 0.060 | [-0.004 ; -0.00008] |
| **Material resources (std) – Explained component** | 0.0033 | 0.0011 | 3.02 | 0.002 | [0.001 ; 0.005] |
| **Classroom size – Explained component** | -0.0013 | 0.0013 | -1.01 | 0.315 | [-0.004 ; 0.001] |
| **Quality assurance (std) – Explained component** | -0.00008 | 0.0003 | -0.28 | 0.780 | [-0.0007 ; 0.0005] |
| **School cultural activities (std) – Explained component** | -0.0009 | 0.0053 | -0.17 | 0.865 | [-0.011 ; 0.009] |
| **Ability grouping (std) – Explained component** | -0.006 | 0.0013 | -4.50 | 0.000 | [-0.008 ; -0.003] |
| **Extra instructional hours (std) – Explained component** | -0.0016 | 0.0012 | -1.40 | 0.161 | [-0.004 ; 0.0006] |
| Student ESCS | -0.0001 | 0.0006 | -0.24 | 0.808 | [-0.001 ; 0.001] |
| Female (*ref*. Males) | -0.0002 | 0.0004 | -0.47 | 0.636 | [-0.001 ; 0.0006] |
| Migration (Mixed, *ref*. Non-migrants) | -0.00004 | 0.0001 | -0.35 | 0.725 | [-0.0002 ; 0.0002] |
| Migration (2^nd^ generation, *ref*. Non-migrants) | 0.00003 | 0.0001 | 0.29 | 0.771 | [-0.0001 ; 0.0002] |
| Migration (1^st^ generation, *ref*. Non-migrants) | -0.0000 | 0.0001 | 0.02 | 0.987 | [-0.0001 ; 0.0001] |
| Language achievement in Grade 8 | -0.004 | 0.0126 | -0.31 | 0.756 | [-0.028 ; 0.021] |
| **Average classroom ESCS – Unexplained component** | -0.031 | 0.01676 | -1.87 | 0.061 | [-0.064 ; 0.001] |
| **Classroom average prior language achievement – Unexplained component** | 0.005 | 0.0056 | -1.87 | 0.061 | [-0.06 ; 0.001] |
| **Challenging behavioural climate (std) – Unexplained component** | -0.001 | 0.0015 | -0.97 | 0.333 | [-0.004 ; 0.001] |
| **Positive disciplinary climate (std) – Unexplained component** | 0.005 | 0.0026 | 1.84 | 0.065 | [-0.0003 ; 0.0101] |
| **Low teacher support (std) – Unexplained component** | 0.0002 | 0.0008 | 0.30 | 0.761 | [-0.0014 ; 0.002] |
| **Effective classroom management (std) – Unexplained component** | -0.0007 | 0.0005 | -1.37 | 0.171 | [-0.002 ; 0.0003] |
| **Instructional adaptivity – Unexplained component** | 0.0017 | 0.0014 | 1.21 | 0.226 | [-0.001 ; 0.004] |
| **Positive student-teacher relations (std) – Unexplained component** | -0.00003 | 0.0002 | -0.18 | 0.857 | [-0.0004 ; 0.0003] |
| **Teacher enthusiasm (std) – Unexplained component** | 0.003 | 0.0016 | 1.81 | 0.071 | [-0.0002 ; 0.0066] |
| **Unresponsive teaching – Unexplained component** | -0.008 | 0.0457 | -0.17 | 0.862 | [-0.097 ; 0.081] |
| **Material resources (std) – Unexplained component** | -0.0006 | 0.0007 | -0.82 | 0.413 | [-0.002 ; 0.0008] |
| **Classroom size – Unexplained component** | -0.003 | 0.0825 | -0.03 | 0.973 | [-0.164 ; 0.159] |
| **Quality assurance (std) – Unexplained component** | -0.0003 | 0.0005 | -0.65 | 0.517 | [-0.001 ; 0.0006] |
| **School cultural activities (std) – Unexplained component** | 0.0013 | 0.0017 | 0.78 | 0.434 | [-0.002 ; 0.004] |
| **Ability grouping (std) – Unexplained component** | -0.00006 | 0.0005 | -0.13 | 0.900 | [-0.001 ; 0.0009] |
| **Extra instructional hours (std) – Unexplained component** | -0.0004 | 0.0003 | -1.25 | 0.210 | [-0.001 ; 0.0002] |
| Student ESCS | 0.0007 | 0.0114 | 0.06 | 0.952 | [-0.021 ; 0.023] |
| Female (*ref*. Males) | -0.011 | 0.017 | -0.67 | 0.502 | [-0.044 ; 0.022] |
| Migration (Mixed, *ref*. Non-migrants) | -0.0019 | 0.0033 | -0.58 | 0.564 | [-0.008 ; 0.004] |
| Migration (2^nd^ generation, *ref*. Non-migrants) | -0.003 | 0.0024 | -1.37 | 0.172 | [-0.008 ; 0.001] |
| Migration (1^st^ generation, *ref*. Non-migrants) | -0.003 | 0.0016 | -1.99 | 0.046 | [-0.006 ; 0.0005] |
| Language achievement in Grade 8 | -0.048 | 0.0101 | -4.73 | 0.000 | [-0.067 ; 0.028] |
| Constant | 0.035 | 0.0992 | 0.36 | 0.718 | [-0.158 ; 0.230] |

*Note.* Blinder-Oaxaca decomposition with coefficients from a pooled linear model including a group dummy.
Group 2: Technical track students (N = 10 332); Group 1: Lyceum track students (N = 12 792); total N = 23 124.
Significance levels: * p<0.10, ** p<0.05, *** p<0.01.

Percentage contribution of the explained component to the track-related language gap

| **Variable** | **% explained** | **p-value** |
| --- | --- | --- |
| Average classroom ESCS | 34.28 | 0.000 |
| Classroom average prior language achievement | 80.64 | 0.000 |
| Challenging behavioural climate | -1.97 | 0.339 |
| Positive disciplinary climate | 23.20 | 0.000 |
| Low teacher support | 0.55 | 0.144 |
| Effective classroom management | 0.13 | 0.787 |
| Instructional adaptivity | -0.75 | 0.140 |
| Positive student-teacher relations | 0.227 | 0.364 |
| Teacher enthusiasm | -4.60 | 0.116 |
| Unresponsive teaching | -0.85 | 0.067 |
| Material resources | 1.42 | 0.003 |
| Classroom size | -0.58 | 0.325 |
| Quality assurance | -0.04 | 0.779 |
| Cultural activities | -0.39 | 0.865 |
| Ability grouping | -2.52 | 0.00 |
| Extra instructional hours | -0.71 | 0.163 |

*Note.* Average classroom socioeconomic composition (ESCS) and classroom average prior ability represents **composition**; behavioural and disciplinary climate represent **school climate**; teacher support, classroom management, adaptivity, student-teacher relations, instructional enjoyment, and unresponsive teaching represent **pedagogy**; and material resources, class size, educational resources, cultural activities, ability grouping, and extra instructional hours represent **school resources**. Percentages represent each variable’s contribution to the explained component of the track-related language achievement gap. Positive values increase the explained gap, whereas negative values reduce it. Contributions are expressed relative to the overall explained component and may therefore not sum to 100% because some variables have negative contributions.

Table B6 – Kitagawa-Oaxaca-Blinder decomposition of the lyceums-technical schools net achievement gap (mathematics competencies): contribution of the detailed school-level mediators

| **Component** | **Coefficient** | **Standard Error** | ***Z*** | **P-value** | **95% Confidence Interval** |
| --- | --- | --- | --- | --- | --- |
| Lyceum-track students (g1) | 0.653 | 0.0088 | 74.23 | 0.000 | [0.636 ; 0.670] |
| Technical-track students (g2) | 0.323 | 0.0239 | 13.53 | 0.000 | [0.277 ; 0.371] |
| Difference (g1 – g2) | 0.329 | 0.0255 | 12.91 | 0.000 | [0.279 ; 0.379] |
| Explained component | 0.367 | 0.0278 | 13.22 | 0.000 | [0.313 ; 0.422] |
| Unexplained component | -0.038 | 0.0272 | -1.40 | 0.161 | [-0.091 ; 0.015] |
| **Average classroom ESCS –**  **Explained component** | 0.075 | 0.0183 | 4.10 | 0.000 | [0.039 ; 0.110] |
| **Classroom average prior mathematics achievement – Explained component** | 0.264 | 0.0153 | 17.21 | 0.000 | [0.233 ; 0.293] |
| **Challenging behavioural climate (std) – Explained component** | -0.003 | 0.0062 | -0.55 | 0.581 | [-0.015 ; 0.008] |
| **Positive disciplinary climate (std) – Explained component** | 0.053 | 0.012 | 4.43 | 0.000 | [0.029 ; 0.076] |
| **Low teacher support (std) – Explained component** | 0.003 | 0.0015 | 1.88 | 0.061 | [-0.0001 ; 0.006] |
| **Effective classroom management (std) – Explained component** | 0.0027 | 0.0014 | 1.87 | 0.061 | [-0.0001 ; 0.005] |
| **Instructional adaptivity – Explained component** | -0.0018 | 0.0012 | -1.52 | 0.128 | [-0.004 ; 0.0005] |
| **Positive student-teacher relations (std) – Explained component** | -0.0001 | 0.00046 | -0.31 | 0.756 | [-0.0010 ; 0.0007] |
| **Teacher enthusiasm (std) – Explained component** | -0.033 | 0.0084 | -3.89 | 0.000 | [-0.049 ; -0.016] |
| **Unresponsive teaching – Explained component** | 0.002 | 0.0011 | 1.56 | 0.118 | [-0.0004 ; 0.004] |
| **Material resources (std) – Explained component** | 0.004 | 0.0012 | 3.23 | 0.001 | [0.0016 ; 0.0064] |
| **Classroom size – Explained component** | -0.0014 | 0.0014 | -1.01 | 0.312 | [-0.004 ; 0.001] |
| **Quality assurance (std) – Explained component** | 0.0002 | 0.0003 | 0.66 | 0.511 | [-0.0004 ; 0.0009] |
| **School cultural activities (std) – Explained component** | 0.007 | 0.0052 | 1.29 | 0.197 | [-0.003 ; 0.017] |
| **Ability grouping (std) – Explained component** | -0.006 | 0.0013 | -4.35 | 0.000 | [-0.008 ; -0.003] |
| **Extra instructional hours (std) – Explained component** | 0.0015 | 0.0013 | 1.10 | 0.273 | [-0.001 ; 0.0009] |
| Student ESCS | -0.0001 | 0.0005 | -0.24 | 0.809 | [-0.0012 ; 0.0042] |
| Female (*ref*. Males) | 0.0005 | 0.0010 | 0.48 | 0.628 | [-0.0015 ; 0.0025] |
| Migration (Mixed, *ref*. Non-migrants) | -0.00009 | 0.0002 | -0.42 | 0.676 | [-0.0005 ; 0.0003] |
| Migration (2^nd^ generation, *ref*. Non-migrants) | 0.00003 | 0.00001 | 0.29 | 0.772 | [-0.0002 ; 0.0002] |
| Migration (1^st^ generation, *ref*. Non-migrants) | 0.00004 | 0.0001 | 0.40 | 0.692 | [-0.0002 ; 0.0002] |
| Mathematics achievement in Grade 8 | 0.0004 | 0.0136 | 0.03 | 0.974 | [-0.026; 0.027] |
| **Average classroom ESCS – Unexplained component** | -0.0436 | 0.0191 | -2.29 | 0.022 | [-0.0811 ; -0.0062] |
| **Classroom average prior mathematics achievement – Unexplained component** | 0.025 | 0.0103 | 2.47 | 0.013 | [0.005 ; 0.045] |
| **Challenging behavioural climate (std) – Unexplained component** | 0.0006 | 0.0016 | 0.36 | 0.720 | [-0.0026 ; 0.004] |
| **Positive disciplinary climate (std) – Unexplained component** | 0.009 | 0.0031 | 2.82 | 0.005 | [0.003 ; 0.015] |
| **Low teacher support (std) – Unexplained component** | 0.0003 | 0.0007 | 0.43 | 0.670 | [-0.0011 ; 0.0017] |
| **Effective classroom management (std) – Unexplained component** | -0.0011 | 0.0006 | -1.85 | 0.065 | [-0.0022 ; 0.00006] |
| **Instructional adaptivity – Unexplained component** | 0.0007 | 0.0012 | 0.54 | 0.592 | [-0.0018 ; 0.0031] |
| **Positive student-teacher relations (std) – Unexplained component** | -0.0002 | 0.0002 | -1.03 | 0.301 | [-0.0006 ; 0.0002] |
| **Teacher enthusiasm (std) – Unexplained component** | 0.0045 | 0.0022 | 1.98 | 0.047 | [0.00005 ; 0.0089] |
| **Unresponsive teaching – Unexplained component** | -0.0314 | 0.0534 | -0.59 | 0.557 | [-0.136 ; 0.073] |
| **Material resources (std) – Unexplained component** | -0.0006 | 0.0006 | -0.98 | 0.325 | [-0.0019 ; 0.0006] |
| **Classroom size – Unexplained component** | -0.2202 | 0.0793 | -2.78 | 0.006 | [-0.375 ; -0.064] |
| **Quality assurance (std) – Unexplained component** | -0.00027 | 0.0005 | -0.49 | 0.621 | [-0.0013 ; 0.0008] |
| **School cultural activities (std) – Unexplained component** | 0.0025 | 0.0021 | 1.23 | 0.218 | [-0.0015 ; 0.0066] |
| **Ability grouping (std) – Unexplained component** | 0.0002 | 0.0005 | 0.38 | 0.704 | [-0.0008 ; 0.0011] |
| **Extra instructional hours (std) – Unexplained component** | -0.0008 | 0.0004 | -1.97 | 0.049 | [-0.0016 ; -0.0002] |
| Student ESCS | -0.0010 | 0.0129 | -0.08 | 0.937 | [-0.026 ; 0.024] |
| Female (*ref*. Males) | -0.024 | 0.0188 | -1.28 | 0.199 | [-0.061 ; 0.012] |
| Migration (Mixed, *ref*. Non-migrants) | -0.0016 | 0.003 | -0.54 | 0.592 | [-0.007 ; 0.004] |
| Migration (2^nd^ generation, *ref*. Non-migrants) | -0.0019 | 0.0023 | -0.83 | 0.404 | [-0.006 ; 0.002] |
| Migration (1^st^ generation, *ref*. Non-migrants) | -0.0022 | 0.0013 | -1.72 | 0.085 | [-0.005 ; 0.0003] |
| Mathematics achievement in Grade 8 | -0.033 | 0.0156 | -2.11 | 0.035 | [-0.063 ; -0.002] |
| Constant | 0.281 | 0.0954 | 2.94 | 0.003 | [0.094 ; 0.468] |

*Note.* Blinder-Oaxaca decomposition with coefficients from a pooled linear model including a group dummy.
Group 2: Technical track students (N = 10 332); Group 1: Lyceum track students (N = 12 792); total N = 23 124.
Significance levels: * p<0.10, ** p<0.05, *** p<0.01.

Percentage contribution of the explained component to the track-related mathematics gap

| **Variable** | **% explained** | **p-value** |
| --- | --- | --- |
| Average classroom ESCS | 22.77 | 0.000 |
| Classroom average prior mathematics achievement | 80.11 | 0.000 |
| Challenging behavioural climate | -1.05 | 0.584 |
| Positive disciplinary climate | 16.18 | 0.000 |
| Low teacher support | 0.89 | 0.062 |
| Effective classroom management | 0.83 | 0.060 |
| Instructional adaptivity | -0.55 | 0.131 |
| Positive student-teacher relations | -0.04 | 0.756 |
| Teacher enthusiasm | -9.98 | 0.000 |
| Unresponsive teaching | 0.55 | 0.120 |
| Material resources | 1.22 | 0.002 |
| Class size | -0.44 | 0.320 |
| Quality assurance | 0.07 | 0.511 |
| Cultural activities | 2.06 | 0.200 |
| Ability grouping | -1.74 | 0.000 |
| Extra instructional hours | 0.46 | 0.278 |

*Note.* Average classroom socioeconomic composition (ESCS) and classroom average prior ability represents **composition**; behavioural and disciplinary climate represent **school climate**; teacher support, classroom management, adaptivity, student-teacher relations, instructional enjoyment, and unresponsive teaching represent **pedagogy**; and material resources, class size, educational resources, cultural activities, ability grouping, and extra instructional hours represent **school resources**. Percentages represent each variable’s contribution to the explained component of the track-related mathematics achievement gap. Positive values increase the explained gap, whereas negative values reduce it. Contributions are expressed relative to the overall explained component and may therefore not sum to 100% because some variables have negative contributions.
